# *Oryza sativa* L. Indica Seed Coat Ameliorated Concanavalin A—Induced Acute Hepatitis in Mice via MDM2/p53 and PKCα/MAPK1 Signaling Pathways

**DOI:** 10.3390/ijms241914503

**Published:** 2023-09-25

**Authors:** Zhiye Zhao, Ye Li, Shancheng Guo, Yuxu Chen, Haiaolong Yin, Yaxian Li, Guiguang Cheng, Lei Tian

**Affiliations:** 1 Faculty of Food Science and Engineering, Kunming University of Science and Technology, Kunming 650500, China; 2 School of Basic Medicine, Kunming University of Science and Technology, Kunming 650500, China; liye918@gmail.com

**Keywords:** *Oryza sativa* L. indica, acute hepatitis, MDM2, PKCα, PTGS1, gut microbiota

## Abstract

Acute hepatitis (AH) is a common liver disease with an increasing number of patients each year, requiring the development of new treatments. Hence, our work aimed to evaluate the therapeutic effect of *Oryza sativa* L. indica (purple rice) seed coat on concanavalin A (ConA)-induced AH and further reveal its potential mechanisms. Purple rice seed coat extract (PRE) was extracted with hydrochloric acid ethanol and analyzed through a widely targeted components method. We evaluated the effects of PRE on AH through histopathological examination, liver function, gut microbiota composition, and the intestinal barrier. The potential targets of PRE on AH were predicted by bioinformatics. Western blotting, terminal deoxynucleotidyl transferase-mediated dUTP-biotin nick end labeling assay (TUNEL) staining, and corresponding kits were used to investigate PRE effects on predicting targets and associated signaling pathways in AH mice. In AH model mice, PRE treatment increased transformed mouse 3T3 cell double minute 2 (MDM2) expression to inhibit apoptosis; it also markedly downregulated protein kinase C alpha (PKCα), prostaglandin-endoperoxide synthase 1 (PTGS1), and mitogen-activated protein kinase 1 (MAPK1) activity to alleviate inflammation. Thus, PRE treatment also recovered the intestinal barrier, decreased the lipopolysaccharide (LPS) levels of plasma and the liver, enhanced liver function, and improved the composition of intestinal microbiota. In general, PRE targeting MDM2, PKCα, MAPK1, and PTGS1 ameliorated ConA-induced AH by attenuating inflammation and apoptosis, restoring the intestinal barrier, enhancing the liver function, and improving the gut microbiota, which revealed that the purple rice seed coat might hold possibilities as a therapeutic option for AH.

## 1. Introduction

AH is a typical life-threatening liver disease, causing liver dysfunction that can progress to acute liver failure (ALF) [1], which seriously endangers people’s health in the world [2,3]. It is caused by various reasons, usually viruses or drugs, and has a high mortality rate [4,5]. ALF can induce local and systemic syndromes due to the immune response and hepatic cell death, which are referred to as the most typical reasons for mortality [6]. The liver, as the main metabolic organ, can decompose and clean toxins to reduce the toxin damage to the body [7]. Despite its physiological protecting process, the liver is also susceptible to impairments from various toxins or compound metabolites. AH-exacerbated progression may lead to the occurrence of acute liver failure [8]. So, the prevention and treatment of acute hepatitis have always been a research focus [9]. The ConA-induced acute hepatitis mouse model is a widely accepted animal model that can simulate acute hepatitis and liver injuries and is remembered as an appropriate model of viral hepatitis, autoimmune hepatitis, and related acute liver failure [10]. ConA-induced acute hepatitis results in the production of various hepatotoxic cytokines, the death of hepatocytes, oxidative stress, and inflammatory damage [11]. Liver inflammatory cytokines take part in the pathogenesis of hepatitis; tumor necrosis factor alpha (TNF-α) is one important factor that triggers hepatocyte apoptosis [12].

*Oryza sativa* L. indica is a type of grass rice and is classified as indica rice, which is close to wild rice and related to japonica rice. It is usually known as purple rice because of its brownish-purple seed coat [13]. Previous studies have revealed that purple rice has various physiological functions such as improving iron deficiency anemia, scavenging free radicals, delaying aging, anti-stress responses, and immune regulation [14], and the active ingredients of purple rice are mainly composed of flavonoids, alkaloids, phenols, etc. [15]. The antioxidant capacity of purple rice is remarkably stronger than that of white rice because the flavonoid contents are substantial in purple rice. Moreover, purple rice can modulate the gut microbiota composition to improve colitis [16]. The effect of purple rice on AH and its corresponding mechanism remains unclear as yet. The main purpose of this research was to investigate the role of PRE on ConA-induced AH and its underlying mechanisms. In this study, we extracted the seed coat of purple rice with hydrochloric acid ethanol and analyzed PRE using a widely targeted components approach. Through a bioinformatics analysis and experimental validation, we found that PRE could protect mice against ConA-induced AH. We also demonstrated regulatory mechanisms where the PRE treatment ameliorated ConA-induced AH by regulating MDM2/p53 and PKCα/MAPK1 pathways, attenuating inflammation and apoptosis, restoring the intestinal barrier, enhancing the liver function, and improving the gut microbiota in vivo.

## 2. Results

### 2.1. Extraction and Identification of Seed Coat Components of Oryza sativa L. Indica

**Figure 1:**
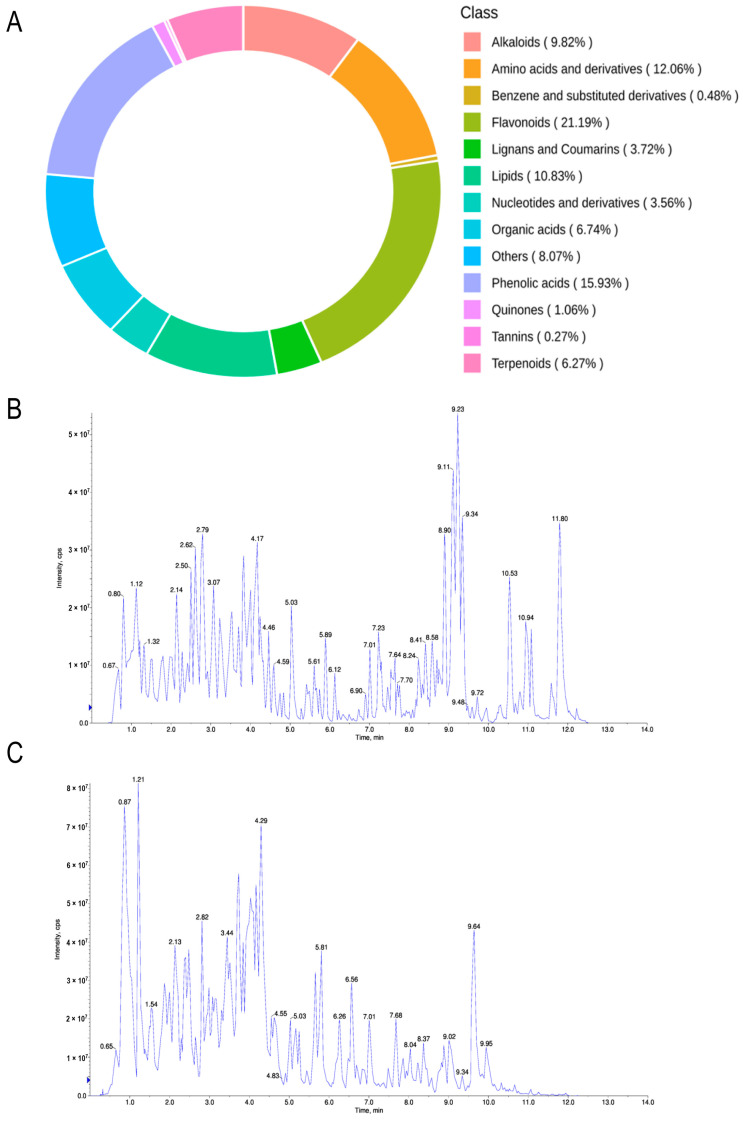
Various components were identified in PRE by widely targeted components analysis. (**A**) PRE components were identified with widely targeted components analysis. (**B**) The total ion current diagram of PRE samples in positive ionization mode. (**C**) The total ion current diagram of PRE samples in negative ionization mode.

To identify the PRE chemical compositions, PRE was performed using a widely targeted components analysis (Figure 1A). A total of 1525 components were identified using the Metware database, of which flavonoids accounted for 21.19%, phenolic acids accounted for 15.93%, alkaloids accounted for 9.82%, amino acids and their derivatives accounted for 12.06%, lipids accounted for 10.83%, organic acids accounted for 6.74%, tannins accounted for 6.27%, lignans and coumarin accounted for 3.72%, nucleotides and their derivatives accounted for 3.56%, quinones accounted for 1.06%, benzene and its substituted derivatives accounted for 0.48%, terpenoids accounted for 0.27% and others accounted for 8.07%. The total ion current diagrams of PRE samples in positive (Figure 1B) and negative (Figure 1C) ionization modes are shown.

### 2.2. PRE Improved Pathological Liver Injury and Liver Function in AH Mice

**Figure 2:**
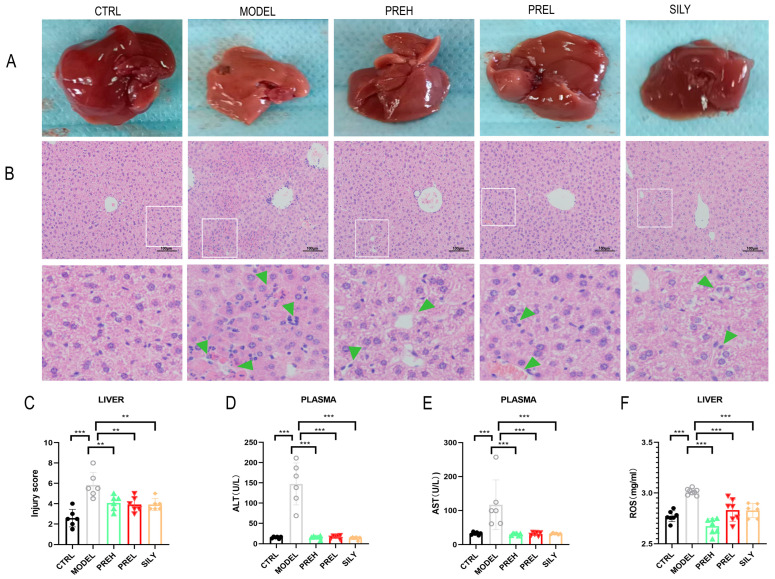
PRE improved liver pathological injury and liver function in AH mice. (**A**) Effect of PRE treatment on liver morphology. **(B**) Livers’ hematoxylin and eosin (HE) staining: the white frames were extended and shown above, and green arrows point to the lesions. Scaleline was 100 μm. (**C**) The injury score of livers. (**D**) Levels of ALT in plasma. (**E**) Levels of AST in plasma. (**F**) Levels of ROS in hepatic tissues. Data are presented as mean ± standard error of mean (SEM), *n* = 6. ** *p* < 0.01, *** *p* < 0.001.

The livers of the control (CTRL) group were reddish-brown, bright in color, and with soft texture, and the livers of the AH Model group were dark yellow and had obvious lesions, but the livers of the PRE high dose (PREH), PRE low dose (PREL), and silymarin (SILY) groups were significantly improved (Figure 2A). HE staining of the livers showed that PRE treatment alleviated the liver’s pathological injury in AH mice, which was identical to the SILY treatment (Figure 2B). The liver injury score of the Model group was higher than that of the CTRL group, while the liver injury scores of the PREH, PREL, and SILY groups were lower than that of the Model group (Figure 2C). In the Model group, the levels of alanine aminotransferase (ALT) and aspartate aminotransferase (AST) in the plasma were increased, and their levels were lower in the PREH, PREL, and SILY groups than in the Model group (Figure 2D,E). The reactive oxygen species (ROS) level of livers in the Model group was higher than the CTRL group, but ROS levels of PREH, PREL, and SILY were significantly decreased (Figure 2F). The results indicated that PRE reduced the levels of AST, ALT, and ROS and improved liver function in AH mice.

### 2.3. PRE May Ameliorate AH by Regulating Target Genes as Predicted by Bioinformatics

**Figure 3:**
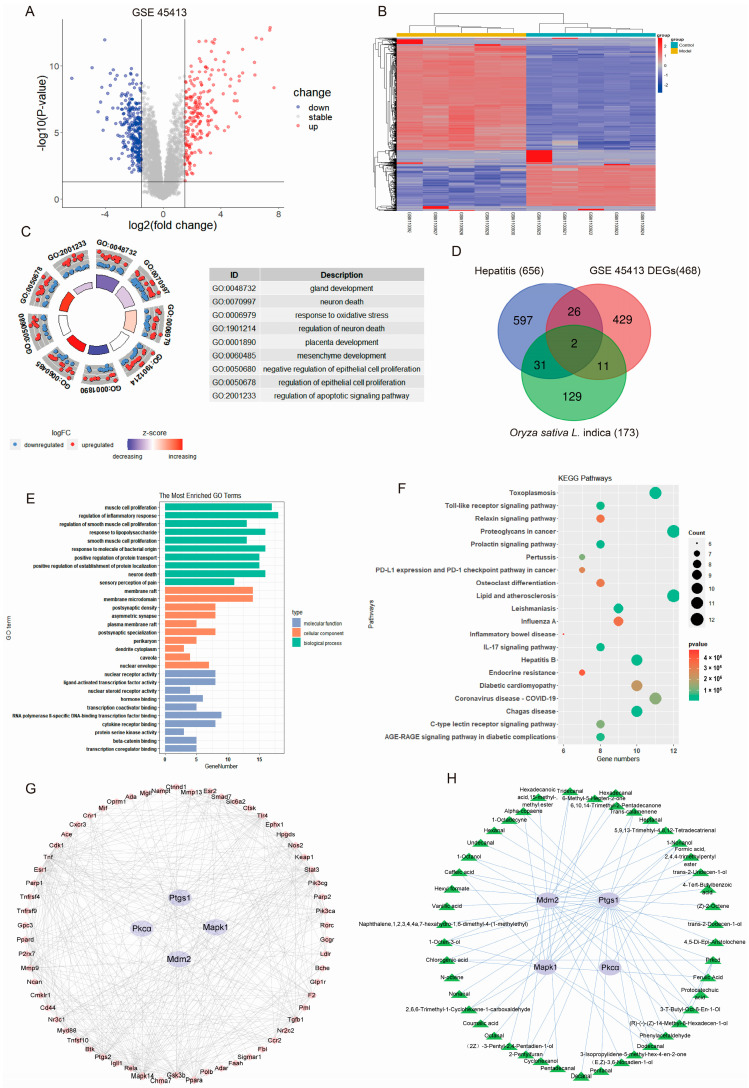
Exploration and identification of potential targets in AH regulated by *Oryza sativa* L. indica. (**A**) Volcano plots of DEGs between AH and normal livers in GSE45413, red plots present upregulated genes, blue plots present downregulated genes, and gray plots present stable genes (*p* < 0.05). (**B**) DEGs’ heatmap between AH and normal liver in GSE45413. (**C**) GO analysis. The top 9 enriched DEG terms. (**D**) Venn diagram. AH-related genes, DEGs of GSE45413, and potential target genes of *Oryza sativa* L. indica. (**E**) The molecular function, cellular components, and biological process-enriched terms were analyzed by hub gene GO analysis. (**F**) KEGG pathway prediction with hub genes showed the top 20 relevant pathways. (**G**) PPI network. The red and purple plots present hub genes, and the edges present their communication. (**H**) Pharmacological target and compound network of *Oryza sativa* L. indica against AH. The purple plots present the targets, the green plots present the compounds, and the edges present their interplay.

In GSE45413, 468 differentially expressed genes (DEGs) of AH were obtained, including 262 downregulated and 206 upregulated genes (Figure 3A). There is a significant difference in DEGs between the normal control group and the AH group of GSE45413 (Figure 3B). The top nine terms of DEG ontology (GO) annotation are shown in Figure 3C. We picked out 38 main compounds of PRE in two databases (Table S1). Afterward, 173 potential targets and the related 38 compounds were obtained in the Swiss Target database (Table S2), and 656 AH-associated genes were retrieved in DisGeNET (Table S3). A total of 70 hub genes were filtrated among AH-associated genes, potential *Oryza sativa* L. indica target genes, and DEGs of GSE45413 (Figure 3D). Next, the exploration and enrichment of overlapping genes from three domains, including molecular function, cellular components, and biological processes, were performed by GO analysis annotation and obtained the top 10 of them (Figure 3E). The top 20 Kyoto Encyclopedia of Genes and Genomes (KEGG) pathways were revealed in Figure 3F, including hepatitis B, diabetic cardiomyopathy, and so on. Hub genes’ PPI was ascertained by STRING, and Cytoscape was used to construct the network, which consisted of 66 nodes and 391 edges (Figure 3G). In addition, an *Oryza sativa* L. indica compound and target network of PRE against AH was constructed (Figure 3H). Taken together with the results of the above analysis and the literature reports, MDM2, PTGS1, PKCα, and MAPK1 were chosen as prospective targets.

### 2.4. PRE Attenuated Liver Apoptosis in AH Mice through Upregulating MDM2

**Figure 4:**
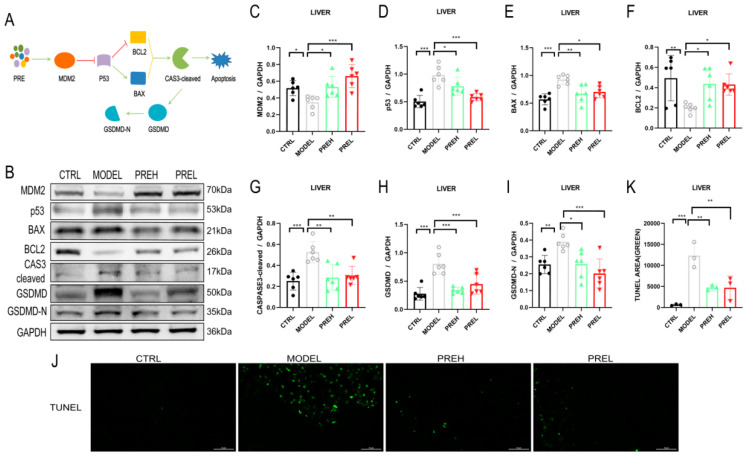
PRE inhibited apoptosis in AH model by upregulating MDM2. (**A**) Illustration of PRE suppressing AH apoptosis by MDM2. (**B**–**I**) The protein expressions of MDM2, P53, BCL2, BAX, CASP3-cleaved, GSDMD, and GSDMD-N in mice livers. (**J**,**K**) Representative TUNEL staining images were quantified by TUNEL-positive cells in indicated groups. Scale bars were 100 μm. Data are presented as mean ± SEM, *n* = 6. * *p* < 0.05, ** *p* < 0.01, and *** *p* < 0.001.

Previous research studies reported that p53 regulated apoptosis via BCL2-Associated X (BAX) [17,18,19] and B cell leukemia/lymphoma 2 (BCL2) [20,21,22]. To confirm the effect of PRE on apoptosis by the MDM2/p53 pathway in AH mice, the levels of associated factors were investigated in the liver. In the AH model, MDM2 and BCL2 levels were downregulated, and PREH and PREL treatment upregulated them (Figure 4C,F). The expression of p53, BAX, and CASPASE3 (CAS3) -cleaved were all increased in AH mice; comparatively, PREH and PREL treatment sharply reduced them (Figure 4D,E,G). Some research also found that CASPASE3-cleaved induced GSDMD-N boosting to stimulate apoptosis [23]. Afterward, the levels of gasdermin D (GSDMD) and GSDMD-N were enhanced in the AH model, and PREH and PREL treatment decreased GSDMD and GSDMD-N (Figure 4H,I). At the same time, we found fewer TUNEL-positive cells in the PREH and PREL groups compared with the MODEL group (Figure 4J,K). Taken together, PRE treatment restrained apoptosis in AH mice livers by upregulating MDM2.

### 2.5. PRE Alleviated Inflammation in AH Mice through Downregulating PTGS1 and PKCα

**Figure 5:**
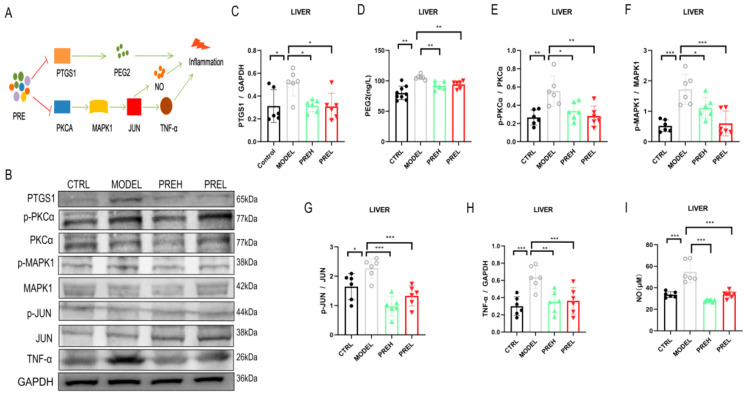
PRE alleviated inflammation in AH mice through downregulating PTGS1 and PKCα. (**A**) Illustration of PRE disincentiving AH inflammation by downregulating PTGS1 and PKCα. (**B**,**C**,**E**–**H**) Western blot analyses of PTGS1, PKCα phosphorylation, MAPK1 phosphorylation, JUN phosphorylation, and TNF-α levels in livers. (**D**) Levels of PEG2 in hepatic tissues. (**I**) Levels of NO in hepatic tissues. Data are presented as mean ± SEM, *n* = 6. * *p* < 0.05, ** *p* < 0.01, and *** *p* < 0.001.

In order to study the effects of PRE on inflammation via PTGS1 and PKCα, the levels of pathway factors were detected in the liver tissues. In the AH mouse model, PTGS1 and TNF-α expressions were significantly upregulated; administration of PREH and PREL both remarkably downregulated them (Figure 5C,H). The phosphorylation levels of PKCα, MAPK1 and jun proto-oncogene (JUN) were obviously increased in the AH Model; however, the PREH and PREL treatment could remarkably decrease them all (Figure 5E–G). Similarly, in the Model group, prostaglandin E2 (PGE2) and nitric oxide (NO) levels were raised, but PREH and PREL treatment both reduced them (Figure 5D, I). Taken together, PRE attenuated the inflammation in AH mice via attenuating the PTGS1/PGE2 and PKCα/JUN signaling pathways.

### 2.6. PRE Improved the Intestinal Barrier and Downregulated LPS Levels in AH Mice

**Figure 6:**
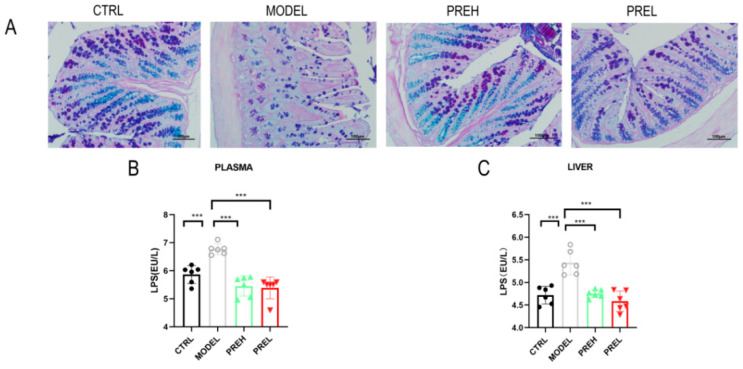
PRE improved intestinal barrier and downregulated LPS levels in AH mice. (**A**) AB-PBS staining of mice colon. Scale bars were 100 μm. (**B**) LPS concentrations of plasma. (**C**) LPS concentrations of hepatic tissue. Data are presented as mean ± SEM, *n* = 6. *** *p* < 0.001.

By analyzing the alcian blue (AB)-periodic acid Schiff (PAS) staining pattern of the colon, we found that the mucin density was significantly reduced and the mucin arrangement was destroyed in AH model mice, and then the PRE treatment restored them (Figure 6A). In the AH Model, the levels of LPS in the plasma and liver tissues increased significantly, but administration of PREH and PREL all decreased them significantly (Figure 6B,C). 

### 2.7. Effect of PRE on Gut Microbiota in AH Mice

**Figure 7:**
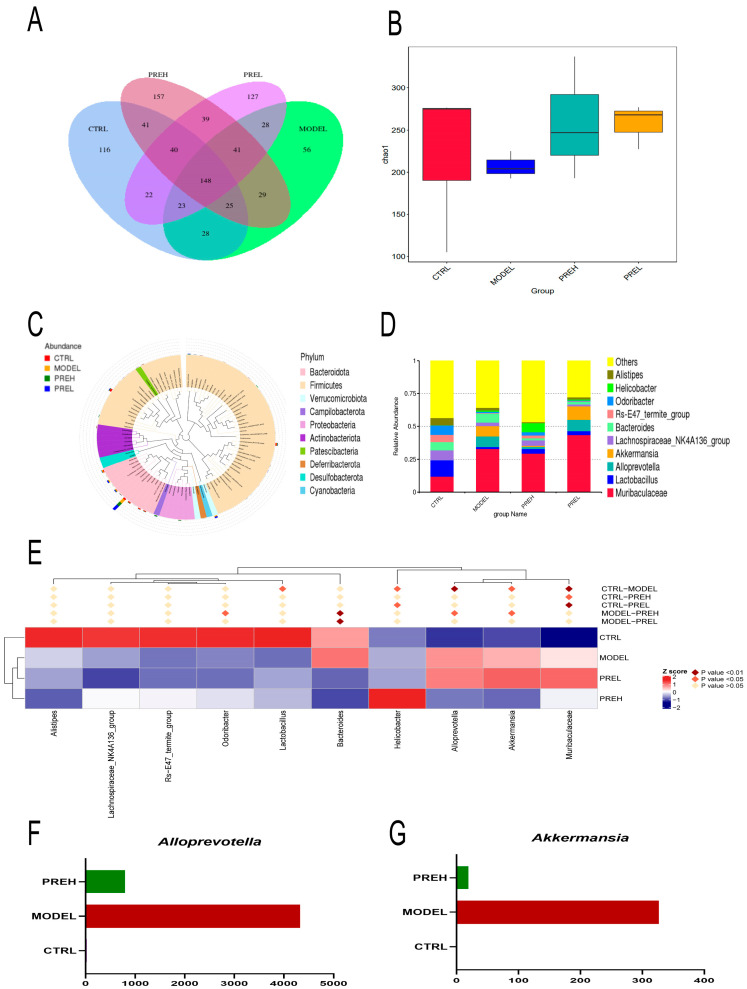
Effect of PRE on the gut microbiota of AH mice. (**A**) Venn diagram illustrating the expression of OTUs in each group. (**B**) Chao 1 indices (α-Diversity indices). (**C**) Relative abundance of gut microbiota at the phylum level (Top 10). (**D**) Relative abundance of gut microbiota at the genus level (Top 10). (**E**) Clustering heatmap of species abundance at genus levels. (**F**,**G**) Relative abundances of *Alloprevotella* and *Akkermansia*, *n* = 3.

We analyzed the microbial community 16s rDNA in mouse feces so as to explore the additional mechanism of PRE action on AH model mice. As shown in the Venn diagram (Figure 7A), there were 148 operational taxonomic units (OTUs) for all samples. In addition, 116, 56, 157, and 127 unique OTUs were identified in CTRL, MODEL, PREH, and PREL groups in AH mice, respectively. The results showed that the Chao-1 index decreased in the MODEL group, and PRE treatment restored these trends (Figure 7B). The top 10 kinds of intestinal bacteria at the genus level are shown in Figure 7C. The results of the top 10 intestinal bacteria at phyla are shown in Figure 7D by evolution tree. In order to identify the bacteria that play an important role in AH mice after PRE administration, the MetaStat method was performed at genus levels (Figure 7E). As it turned out, *Alloprevotella* and *Akkermansia* were significantly decreased after PREH treatment (Figure 7F,G). According to the previous reports, *Alloprevotella* and *Akkermansia* were closely related to the intestinal barrier and diseases. In general, PRE improved the composition of gut microbiota in AH mice.

## 3. Discussion

AH, induced by drugs, toxins, infections, or alcohol with a high incidence rate, is a worldwide health problem [24,25]. Although there are lots of AH studies, it is difficult to reduce mortality with existing treatments [26]. The aim of our study was to find natural products from rice indicators that could alleviate AH as therapeutic agents. We extracted and identified 1525 compounds from PRE, which were rich in active substances, such as flavonoids, phenolic acids, and so on. According to previous reports, flavonoids of plants have anti-inflammatory capacity and antioxidant properties [27,28]. Rice extract significantly improved the physiological function of the liver by inhibiting cell apoptosis, reducing inflammation, and improving gut microbiota in vivo [29,30]. Hence, it is necessary to make a further study about the PRE’s roles on AH.

With the assistance of various bioinformatics methods, the DEGs of AH were identified in GSE45413, which involved 262 downregulated and 206 upregulated mRNAs. A total of 173 predictive molecular targets might respond to the *Oryza sativa L*. indica seed coat, and 656 AH-associated genes were obtained. The molecular function, cellular signaling pathways, and biological processes were performed by GO enrichment analysis methods and KEGG analysis. In gene expression levels with GO and KEGG, inflammation and apoptosis were mainly involved in the AH process. A network of hub gene PPI and a target vs. compound network were constructed to further illustrate interaction modes. TNF-α, as a notable inflammatory factor, helped to understand inflammatory reactions [31]. By comprehensive analysis, we focused on MDM2, PTGS1, PKCα, and MAPK1 as potentially critical actors linked to *Oryza sativa L.* indica in the development of AH. MDM2 could bind to p53 and form a complex, then play a key role in the regulation of p53 stability and inducing cell death, and also be involved in liver injury [32,33]. PTGS1 had both cyclooxygenase and peroxidase activities and regulated oxidative stress and inflammation involved in drug-induced liver injury, non-alcoholic fatty liver disease, and other liver diseases [34]. PKCα dependently activating MAPK pathways could induce TNF-α expression during liver injury, which was an important subject in managing acute inflammatory diseases [35,36]. Our findings suggest that hepatocyte apoptosis, oxidative stress, and inflammation of AH were attenuated by PRE treatment, so we further investigated the potential mechanism in the AH mouse model in vivo.

Apoptosis is an active and procedural death process after cells are regulated by specific genes, and it is also a normal physiological response of cells [37]. We demonstrated that PRE inhibited the apoptosis in AH models. PRE could upregulate the expression of MDM2 and BCL2 and downregulate the expression of p53, GSDMD, and GSDMD-N in AH mice. MDM2 is one of the important molecules that inhibit p53 activity, and their interactions have important functions for cell survival [38]. Meanwhile, TUNEL-positive cells and apoptotic-related proteins BAX and CASP3-cleaved were significantly decreased with PRE treatment in AH mice. These results confirmed that PRE can reduce liver cell apoptosis via the MDM2/p53 signaling pathway in AH mice.

In AH, a serious inflammatory response is also a significant physiologic and pathologic mechanism [39]. PTGS1 could regulate the expression of PGE2 and promote the prostaglandins as an immune response in liver injury [40]. The results showed that PRE could decrease AST and ALT of the plasma in AH mice and NO and PGE2 levels in the AH mice hepatic tissues. Similarly, PRE treatment decreased injury scores and relieved the pathomorphology in AH liver tissues. The regulation networks were that PRE treatment decreased PTGS1 and TNF-α levels and also declined phosphorylation degrees of PKCα, MAPK1, and JUN in AH models. Our outcomes showed that PRE relieved inflammation of AH mice via PTGS1 and PKCα/MAPK1 pathways, and it also supported the elimination of intracellular oxidative stress and the reduction in the generation of ROS [32]. In sum, PRE can alleviate inflammation and improve the antioxidant capacity of the liver in AH mice.

According to previous reports, the LPS infiltrated the plasma via the intestinal barrier in AH mice, and then the plasma LPS induced inflammatory responses and apoptosis [41]. Our data showed that PRE treatment decreased the LPS of the plasma and liver in the AH mice. Moreover, the PRE treatment improved mucin density and the degree of mucin alignment, then enhanced intestinal barrier function. In addition, changes in intestinal flora were detected. Based on the reports, an increase in the *Alloprevotella* abundance in the colon might aggravate inflammation [42,43]. Although *Akkermansia* was generally considered a biomarker of healthy intestines, studies also found that *Akkermansia* was a gram-negative bacteria that could destroy intestinal mucosal barriers and cause inflammation. The abundance of *Akkermansia* was inversely correlated with the expression of NOD-like receptor family pyrin domain containing 6 (NLRP6), an innate immune receptor that protects animals from intestinal injury [44,45]. We observed that gut microbiota abundance changed significantly at the genus level, especially *Alloprevotella* and *Akkermansia*. Compared with the CTRL group, the relative abundance of *Alloprevotella* [46] and *Akkermansia* [47] increased in the MODEL group, and PREH treatment reduced *Alloprevotella* and *Akkermansia*. These results suggested that PRE treatment restored the intestinal barrier and improved gut microbiota in AH mice.

## 4. Materials and Methods

### 4.1. Acquisition and Analysis of AH Differentially Expressed Genes (DEGs)

The AH and normal liver tissue gene expression profiling by array were picked up in GEO (http://www.ncbi.nlm.nih.gov/geo/, accessed on 5 November 2021) [48]. We collected 5 ConA-AH cases and 5 controls from GSE45413 dataset. To single out the AH DEGs, we applied R package dplyr and limma to analyze GSE45413 dataset. To analyze the DEG gene GO, we used R package ggplot2 and clusterProfiler.

### 4.2. Potential Target Prediction of Oryza sativa L. Indica and AH

The main compounds of *Oryza sativa* L. indica were chosen in PubChem or CAS SciFinder databases. We retrieved and screened the related targets of *Oryza sativa* L. indica compounds from Swiss Target Prediction databases [49]. Then, the AH-associated genes were screened from DisGeNET database [50]. We screened hub genes among the targets of *Oryza sativa* L. indica, DEGs of GSE45413, and AH-associated genes with corresponding R packages. KEGG was used to analyze the potential cellular signaling pathways associated with hub genes, and GO was used to visualize the molecular functions, cellular components, and biological processes of hub genes. KEGG and GO visualized analyses were operated with R program. Protein-protein interaction (PPI) of hub genes was ascertained by STRING database [51], and Cytoscape (3.8.0) was used to construct the interaction network. The *Oryza sativa* L. indica compound and target network of purple rice seed coat against AH was built with Cytoscape (3.8.0) as well.

### 4.3. Extraction of Oryza sativa L. Indica Seed Coat

*Oryza sativa* L. indica was purchased from Mojiang, Yunnan Province. It was extracted based on the content with some modifications [52]. In brief, the PRE powder was filtered with an 80-mesh sieve and extracted 3 times with 70% acidified ethanol containing 1% (*v*/*v*) HCl by sonication-assisted method, for 0.5 h each time, in 1:15 ratio of material/solution, following by 1500 g centrifugation for 10 min. Finally, the extract was obtained via evaporation and vacuum lyophilized in the supernatant.

### 4.4. Widely Targeted Components Analysis of PRE

Extensively targeted component analysis is a novel technology that is distinct from existing component detecting processes as it has the multifunction of non-targeted components and the correctness of focused components. It acquires data by triple quadrupole mass (QQQ) spectrometry in multi-reaction monitoring (MRM) mode, which can evaluate many known components and many unidentified components with strong throughput, sensibility, qualitative accuracy, repeatability, and complete database availability. PRE components were identified based on related public databases and local self-built MWDB databases (Metware Biotechnology Co., Ltd., Wuhan, China).

### 4.5. Animal Study

C57BL/6J male mice (7 weeks old) were acquired from Beijing SPF Animal Technology Co., Ltd. (SPF, Beijing, China). All the animals were acclimated for 7 days before the experiment, and the experimental diets and water were available ad libitum. Design and treatment of animal experiments refer to our early research on the natural products. Mice were randomized to the CTRL, MODEL, PREH, PREL, and SILY groups (6 mice/group). CTRL received blank (purified water). PREH and PREL groups were 0.6 g/kg or 0.2 g/kg in weight PRE gavage, individually (PREH and PREL were safe for mice). Meanwhile, SILY group was injected with 0.01 g/kg (weight) silymarin (SILY) (5304299-1g, Aladdin, Shanghai, China). A total of 14 d later, except for the CTRL, AH models in all the other groups were induced via intravenous injection with 0.01 g/kg (weight) ConA (IC4870, Solarbio, Beijing, China) [53]. After 12 h, the mice were sacrificed under isoflurane anesthesia, and then the plasma, livers, colons, and feces were sampled. All studies were processed in accordance with the Institutional Guidelines and Animal Ordinance of Kunming University of Science and Technology Animal Ethical Committee (PZWH (Dian) K2023-0025).

### 4.6. Histological Analysis

The fixed livers were enclosed and evaporated in paraffin to prepare 5 μm paraffin sections. Hematoxylin and eosin (HE) staining was performed after deparaffinization, hydration, and so on. Pathological changes in the liver tissues were observed, and the liver injury scores were estimated [54]. Meanwhile, the paraffin sections of colon tissues were prepared. After dewaxing, hydration, and other steps, sections were flushed for 2 min after 15 min of alcian blue staining, twice again with distilled water after 30 min of periodic acid Schiff staining, and rinsed with water for 5 min; the sheet was then dried and sealed. The colon sections were observed by an optical microscope (Olympus, Shinjuku-ku, Tokyo, Japan).

### 4.7. Detection of Cytokines 

The contents or activities of NO (S0021, Beyotime, Shanghai, China), AST (C010-2-1, Nanjing Jiancheng Bioengineering Institute, Nanjing, Jiangsu, China), and ALT (C009-2-1, Nanjing Jiancheng Bioengineering Institute, Nanjing, Jiangsu, China) were evaluated via kits. The levels of ROS (S0033S, Beyotime, Shanghai, China), PGE2 (YJ028719, Beyotime, Shanghai, China), and LPS (YJ037221, Beyotime, Shanghai, China) in liver tissues were detected by enzyme-linked immunosorbent assay (ELISA) kits. Data were obtained with a spectrophotometer (Biotek, Shoreline, WA, USA).

### 4.8. TUNEL Staining

The mouse liver tissues were rapidly harvested and embedded in the optimal cutting temperature compound (OCT) (SAKURA, Chuo-Ku, Tokyo, Japan) reagent and then frozen and sliced with freezing microtome (Leica, Am Leitz-Park, Wetzlar, Germany). Then, the 10μm frozen sections of mouse liver were prepared for TdT-mediated dUTP nick end labeling (TUNEL) staining. According to the manufacturer’s instructions, we used the TUNEL Assay Kit (C710, Beyotime, Shanghai, China) to detect apoptosis in liver tissues [55]. Images were captured with a fluorescence microscope (Nikon, Minato-ku, Tokyo, Japan) and analyzed by Image Pro Plus (6.0) (Rockville, MD, USA).

### 4.9. 16S rRNA Sequencing of the Gut Microbiota

The mouse fecal samples were collected from four groups (CTRL, MODEL, PREH, and PREL). Bacterial DNA (deoxyribonucleic acid) was extracted from the samples at Novogene Bioinformatics Technology Co. Ltd. (Tianjin, China). Diluted DNA was then used to amplify the 16S rDNA V3-V4 region with barcoded primers. Subsequently, polymerase chain reaction (PCR) products were sequenced and analyzed.

### 4.10. Western Blotting

The mice liver tissues were collected with the RIPA buffer (R0020, Solarbio, Beijing, China). The proteins were detected by bicinchoninic acid (BCA) protein assay kit (P0010, Beyotime, Shanghai, China). Equal protein amounts were loaded for protein electrophoresis. The primary antibodies are listed as follows: MDM2 (1:2000, 66511-1-lg, Proteintech, Wuhan, China), BCL2 (1:2000, 66799-1-lg, Proteintech, Wuhan, China), p53 (1:2000, ab175739, Abcam, Waltham, MA, USA), BAX (1:2000, 50599-2-Ig, Proteintech, Wuhan, China), CASP3 (1:1000, 19677-1-AP, Proteintech, Wuhan, China), GSDMD (1:2000, 20770-1-AP, Proteintech, Wuhan, China), PTGS1 (1:2000, 13393-1-AP, Proteintech, Wuhan, China), Phospho-PKCα (Thr638) (1:1500, 29123-1-AP, Proteintech, Wuhan, China), PKCα (1:1500, 21991-1-AP, Proteintech, Wuhan, China), p-MAPK1 (1:2000, 28733-1-AP, Proteintech, Wuhan, China), MAPK1 (1:2000, 11257-1-AP, Proteintech, Wuhan, China), p-JUN (1:2000, 28891-1-AP, Proteintech, Wuhan, China), JUN (1:2000, 66313-1-Ig, Proteintech, Wuhan, China), TNF-α (1:2000, 26405-1-AP, Proteintech, Wuhan, China), and glyceraldehyde-3-phosphate dehydrogenase (GAPDH) (1:20,000, 60004-1-lg, Proteintech, Wuhan, China). The IgG-horseradish peroxidase (HRP) secondary antibodies (1:10,000, PR30009, Proteintech, Wuhan, China) were incubated. Protein Bands were shown by Novex™ ECL Chemiluminescent Kit (WP20005, Thermo Fisher Scientific, Waltham, MA, USA).

### 4.11. Statistical Analysis

The data were analyzed using one-way analysis of variance (ANOVA), and the differences between the groups were determined using Tukey’s post hoc test. A statistically significant difference was considered as *p* < 0.05, and the data were represented as the mean ± SEM. Statistical analyses were conducted by GraphPad Prism v.8.0.1 (San Diego, CA, USA).

## 5. Conclusions

**Figure 8:**
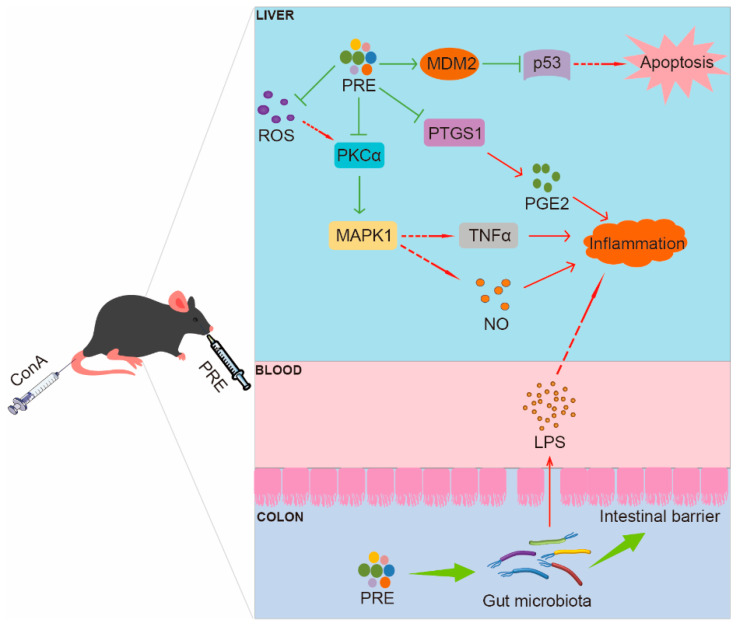
The possible mechanism of *Oryza sativa* L. indica ameliorates acute hepatitis.

In conclusion, our study confirmed that PRE, by targeting MDM2, PKCα, MAPK1, and PTGS1, exhibited ameliorating effects on ConA-induced AH in mice by attenuating inflammation and apoptosis, restoring the intestinal barrier, enhancing liver function, and improving gut microbiota (Figure 8). Consequently, these findings may provide a potential natural rice source for ameliorating AH for pharmaceutical applications.

## Data Availability

The data supporting this study’s findings are available upon request.

## Supplementary Materials

The following supporting information can be downloaded at https://www.mdpi.com/article/10.3390/ijms241914503/s1.
