# Supplementary material for: Oryza sativa L. Indica Seed Coat Ameliorated Concanavalin A—Induced Acute Hepatitis in Mice via MDM2/p53 and PKCα/MAPK1 Signaling Pathways
Source: Int J Mol Sci. 2023 Sep 25;24(19):14503. doi: 10.3390/ijms241914503 (PMC10572155; doi:10.3390/ijms241914503)
Supplement: Supplementary file 1 [file ijms-24-14503-s001.zip › ijms-2608228-supplementary.pdf]

Table S1. Compounds of *Oryza sativa L.* indica. 38 compounds of *Oryza sativa L.* indica were retrieved in CAS SciFinder database and PubChem database.

Compound	CAS ID	Molecular Formula	Molecular Weight	Reference
（2Z）-3-pentyl-2,4-Pentadien-1-ol	149-91-7	C10H18O	154.25	DOI:10.13271/j.mpb.019.006867
（E）-2-Hexenal	6728-26-3	C6H10O	98.14	DOI:10.13271/j.mpb.019.006867
（E）-2-Nonenal	18829-56-6	C9H16O	140.22	DOI:10.13271/j.mpb.019.006867
（E）-2-Octenal	2548-87-0	C8H14O	126.2	DOI:10.13271/j.mpb.019.006867
（E,Z)-3,6-Nonadien-1-ol	1632-73-1	C9H16O	140.22	DOI:10.13271/j.mpb.019.006867
(R)-(-)-(Z)-14-methyl-8-hexadecen-1-ol	481-34-5	C17H34O	254.5	DOI:10.13271/j.mpb.019.006867
(Z)-2-Octene	7642-04-8	C8H16	112.21	DOI:10.13271/j.mpb.019.006867
1-Nonanol	520-18-3	C9H20O	144.25	DOI:10.13271/j.mpb.019.006867
1-Octen-3-ol	527-35-5	C8H16O	128.21	DOI:10.13271/j.mpb.019.006867
2,2,5-Trimethylhexane	3522-94-9	C9H20	128.25	DOI:10.13271/j.mpb.019.006867
2,6,6-trimethyl-1-Cyclohexene-1-carboxaldehyde	432-25-7	C5H10O	152.23	DOI:10.13271/j.mpb.019.006867
3-Isopropylidene-5-methyl-hex-4-en-2-one		C10H16O	152.23	DOI:10.13271/j.mpb.019.006867
3-Methylbutanal	590-86-3	C5H10O	86.13	DOI:10.13271/j.mpb.019.006867
3-t-Butyl-oct-6-en-1-ol		C12H24O	184.32	DOI:10.13271/j.mpb.019.006867
4-tert-Butylbenzoic acid		C11H14O2	178.23	DOI:10.13271/j.mpb.019.006867
5,9,13-trimehtyl-4,8,12-Tetradecatrienal	6040-06-8	C17H28O	248.4	DOI:10.13271/j.mpb.019.006867
6,10,14-trimethyl-2-Pentadecanone	502-69-2	C18H36O	268.5	DOI:10.13271/j.mpb.019.006867
6-methyl-5-Hepten-2-one	110-93-0	C8H14O	126.2	DOI:10.13271/j.mpb.019.006867
7,9-Di-tert-butyl-1-oxaspiro(4,5)deca-6,9-diene-2,8-dione	82304-66-3	C17H24O3	276.4	DOI:10.13271/j.mpb.019.006867
Alpha-copaene	3856-25-5	C15H24	204.35	DOI:10.13271/j.mpb.019.006867
Caffeic acid	331-39-5	C9H8O4	180.16	DOI:10.13652/j.issn.1003-5788.2017.04.006
Chlorogenic acid	327-97-9	C16H18O9	354.31	DOI:10.13652/j.issn.1003-5788.2017.04.006
Cyanidin-3-O-glucoside	7084-24-4	C21H21ClO11	484.8	DOI:10.13652/j.issn.1003-5788.2017.04.006
Cyclohexanol	970-73-0	C6H12O	100.16	DOI:10.13271/j.mpb.019.006867
Dodecanal	112-54-9	C5H10O	184.32	DOI:10.13271/j.mpb.019.006867
Formic acid, 2,4,4-trimethylpentyl ester		C9H18O2	158.24	DOI:10.13271/j.mpb.019.006867
Heptanal	111-71-7	C7H14O	114.19	DOI:10.13271/j.mpb.019.006867
Hexadecanal	629-80-1	C16H32O	240.42	DOI:10.13271/j.mpb.019.006867
Hexadecanoic acid,15-methyl-, methyl ester	6929-04-0	C18H36O2	284.5	DOI:10.13271/j.mpb.019.006867
Pentadecanal	2765-11-9	C6H10O	226.4	DOI:10.13271/j.mpb.019.006867
Protocatechuic acid	99-50-3	C7H6O4	154.12	DOI:10.13652/j.issn.1003-5788.2017.04.006

trans-2-Dodecen-1-ol	126253-42-7	C12H24O	184.32	DOI:10.13271/j.mpb.019.006867
trans-2-Undecen-1-ol	489-41-8	C11H22O	170.29	DOI:10.13271/j.mpb.019.006867
Trans-calamenene	40772-39-2	C15H22	202.33	DOI:10.13271/j.mpb.019.006867
Tridecanal	10486-19-8	C6H12O	198.34	DOI:10.13271/j.mpb.019.006867
Undecanal	112-44-7	C5H10O	170.29	DOI:10.13271/j.mpb.019.006867
Ferulic Acid	537-98-4	C10H10O4	194.18	DOI:10.13652/j.issn.1003-5788.2017.04.006
Vanillic acid	121-34-6	C8H8O4	168.15	DOI:10.13652/j.issn.1003-5788.2017.04.006

Table S2. Potential molecular targets of compounds in *Oryza sativa L.* indica. In Swiss Target Prediction database, 173 potential molecular targets of 38 compounds were collected.

Compound	Target Gene	ChEMBL ID	Uniprot ID	Probability*	Known actives (3D/2D)
Vanillic acid	Akr1c21	CHEMBL1075270	Q91WR5	0.125076	8 / 20
Hexadecanoic acid,15-methyl-, methyl ester	Metap2	CHEMBL1075272	O08663	0.100579	17 / 0
7,9-Di-tert-butyl-1-oxaspiro(4,5)deca-6,9-diene-2,8-dione	Ctsk	CHEMBL1075277	P55097	0.100579	216 / 0
Undecanal	Ephx1	CHEMBL1075293	Q9D379	0.116739	33 / 0
（2Z）-3-pentyl-2,4-Pentadien-1-ol	Ido1	CHEMBL1075294	P28776	0.043918633	9 / 0
3-t-Butyl-oct-6-en-1-ol	Prkcq	CHEMBL1075295	Q02111	0.071787	12 / 0
3-t-Butyl-oct-6-en-1-ol	Npc1l1	CHEMBL1075296	Q6T3U4	0.071787	0 / 7
7,9-Di-tert-butyl-1-oxaspiro(4,5)deca-6,9-diene-2,8-dione	Slc6a9	CHEMBL1075303	P28571	0.100579	26 / 0
6,10,14-trimethyl-2-Pentadecanone	F2	CHEMBL1075308	P19221	0.112042	59 / 0
（E）-2-Hexenal	Trpa1	CHEMBL1075310	Q8BLA8	0.084974	4/2
Undecanal	Gsk3b	CHEMBL1075321	Q9WV60	0.116739	45 / 0
Cyclohexanol	Gpbar1	CHEMBL1255150	Q80SS6	0.074316	0 / 16
6,10,14-trimethyl-2-Pentadecanone	Rorc	CHEMBL1293231	P51450	0.112042	163 / 0
7,9-Di-tert-butyl-1-oxaspiro(4,5)deca-6,9-diene-2,8-dione	Grm5	CHEMBL1641352	Q3UVX5	0.100579	365 / 0
Hexadecanal	Slc5a1	CHEMBL1744523	Q9QXI6	0.048953	82 / 0
Hexadecanoic acid,15-methyl-, methyl ester	Pde10a	CHEMBL1795126	Q8CA95	0.100579	459 / 0
Caffeic acid	Tlr4	CHEMBL1795167	Q9QUK6	0.071787	0 / 20
Tridecanal	S1pr1	CHEMBL1914262	O08530	0.001	38 / 0
（2Z）-3-pentyl-2,4-Pentadien-1-ol	Hsd17b2	CHEMBL1914270	P51658	0.043918633	58 / 0
Caffeic acid	Mif	CHEMBL1926491	P34884	0.08057	1 / 1
Hexadecanoic acid,15-methyl-, methyl ester	Hsd17b3	CHEMBL1932905	P70385	0.100579	24 / 14
trans-2-Dodecen-1-ol	Npbwr1	CHEMBL1938219	P49681	0.001	1 / 0
Hexadecanal	Lgmn	CHEMBL1949492	O89017	0.001	5 / 0
7,9-Di-tert-butyl-1-oxaspiro(4,5)deca-6,9-diene-2,8-dione	Mapk9	CHEMBL2034797	Q9WTU6	0.100579	41 / 0
7,9-Di-tert-butyl-1-oxaspiro(4,5)deca-6,9-diene-2,8-dione	Pde7a	CHEMBL2040702	P70453	0.100579	53 / 0
7,9-Di-tert-butyl-1-oxaspiro(4,5)deca-6,9-diene-2,8-dione	Adora2a	CHEMBL2115	Q60613	0.100579	173 / 0
Dodecanal	Adora2a	CHEMBL2115	Q60613	0.071787	97 / 0
Alpha-copaene	Ppara	CHEMBL2128	P23204	0.109508	1 / 6
6,10,14-trimethyl-2-Pentadecanone	Tspo	CHEMBL2149	P50637	0.112042	214 / 0
Vanillic acid	Ca13	CHEMBL2186	Q9D6N1	0.141787	2 / 3
6,10,14-trimethyl-2-Pentadecanone	Pik3cg	CHEMBL2189158	Q9JHG7	0.112042	64 / 0
Caffeic acid	Mapk1	CHEMBL2207	P63085	0.071787	0 / 1
Vanillic acid	Mmp9	CHEMBL2214	P41245	0.125076	2 / 0
Vanillic acid	Ca7	CHEMBL2216	Q9ERQ8	0.324628	9 / 28
Heptanal	Ces2c	CHEMBL2217	Q91WG0	0.156518	0 / 11
Ferulic Acid	Adora2b	CHEMBL2237	Q60614	0.031227	0 / 1
7,9-Di-tert-butyl-1-oxaspiro(4,5)deca-6,9-diene-2,8-dione	Pde4b	CHEMBL2272	Q8VBU5	0.100579	57 / 0
Chlorogenic acid	Ednra	CHEMBL2286	Q61614	0.09724	0 / 1
4-tert-Butylbenzoic acid	Ptgdr2	CHEMBL2291	Q9Z2J6	0.071787	20 / 0
4-tert-Butylbenzoic acid	Dao	CHEMBL2331068	P18894	0.071787	2 / 1

（E,Z)-3,6-Nonadien-1-ol	Mapk14	CHEMBL2336	P47811	0.023832743	70 /　0
6-methyl-5-Hepten-2-one	Hdac8	CHEMBL2347	Q8VH37	0.023833	1 /　0
7,9-Di-tert-butyl-1-oxaspiro(4,5)deca-6,9-diene-2,8-dione	Slc6a2	CHEMBL2370	O55192	0.100579	38 /　0
7,9-Di-tert-butyl-1-oxaspiro(4,5)deca-6,9-diene-2,8-dione	Hcrtr1	CHEMBL2434819	P58307	0.100579	394 /　0
Hexadecanoic acid,15-methyl-, methyl ester	Ppard	CHEMBL2458	P35396	0.100579	2 /　7
Vanillic acid	Lck	CHEMBL2480	P06240	0.125076	3 /　2
Caffeic acid	Pik3ca	CHEMBL2499	P42337	0.071787	0 /　1
(R)-(-)-(Z)-14-methyl-8-hexadecen-1-ol	Bche	CHEMBL2528	Q03311	0.062622	1 月 2 日
Chlorogenic acid	Prkca	CHEMBL2567	P20444	0.09724	0 /　198
Hexadecanoic acid,15-methyl-, methyl ester	Abcb1a	CHEMBL2573	P21447	0.100579	10 /　0
3-t-Butyl-oct-6-en-1-ol	Ptgs1	CHEMBL2649	P22437	0.071787	4 /　2
6,10,14-trimethyl-2-Pentadecanone	Alpl	CHEMBL2660	P09242	0.112042	3 /　0
6,10,14-trimethyl-2-Pentadecanone	Tacr1	CHEMBL2668	P30548	0.112042	152 /　0
Trans-calamenene	Cdc25b	CHEMBL2723	P30306	0.116739	8 /　1
Hexadecanoic acid,15-methyl-, methyl ester	Pdgfrb	CHEMBL2749	P05622	0.100579	9 /　0
Hexadecanoic acid,15-methyl-, methyl ester	Hmgcr	CHEMBL2764	Q01237	0.100579	0 /　23
Hexadecanoic acid,15-methyl-, methyl ester	Rara	CHEMBL2792	P11416	0.100579	3 /　0
7,9-Di-tert-butyl-1-oxaspiro(4,5)deca-6,9-diene-2,8-dione	Parp2	CHEMBL2794	O88554	0.100579	27 /　0
1-Octen-3-ol	Slc6a3	CHEMBL2799	Q61327	0.054488	75 /　0
(R)-(-)-(Z)-14-methyl-8-hexadecen-1-ol	Top1	CHEMBL2814	Q04750	0.053556	0 /　2
6,10,14-trimethyl-2-Pentadecanone	Oprm1	CHEMBL2858	P42866	0.112042	56 /　0
7,9-Di-tert-butyl-1-oxaspiro(4,5)deca-6,9-diene-2,8-dione	Hdac6	CHEMBL2878	Q9Z2V5	0.100579	28 /　0
Undecanal	Grm1	CHEMBL2892	P97772	0.125076	9 /　0
7,9-Di-tert-butyl-1-oxaspiro(4,5)deca-6,9-diene-2,8-dione	Dhodh	CHEMBL2991	O35435	0.100579	5 /　0
7,9-Di-tert-butyl-1-oxaspiro(4,5)deca-6,9-diene-2,8-dione	Ace	CHEMBL2994	P09470	0.100579	4 /　0
Caffeic acid	Esr2	CHEMBL2995	O08537	0.150924	1 月 10 日
Dodecanal	Htr2c	CHEMBL3006	P34968	0.071787	57 /　0
Chlorogenic acid	Pygl	CHEMBL3008	Q9ET01	0.09724	15 /　1
（E）-2-Octenal	Slc5a7	CHEMBL3013	Q8BGY9	0.023833	1 /　0
6,10,14-trimethyl-2-Pentadecanone	Cnr1	CHEMBL3037	P47746	0.112042	723 /　0
7,9-Di-tert-butyl-1-oxaspiro(4,5)deca-6,9-diene-2,8-dione	Maob	CHEMBL3050	Q8BW75	0.100579	331 /　0
1-Nonanol	Ar	CHEMBL3056	P19091	0.166712	165 /　9
Cyclohexanol	Esr1	CHEMBL3065	P19785	0.105844	0 /　1
(R)-(-)-(Z)-14-methyl-8-hexadecen-1-ol	Nr1i3	CHEMBL3069	O35627	0.153377	0 /　3
（E）-2-Nonenal	Rxra	CHEMBL3084	P28700	0.001	0 /　11
6,10,14-trimethyl-2-Pentadecanone	Acaca	CHEMBL3086	Q5SWU9	0.112042	75 /　0
Chlorogenic acid	Mmp2	CHEMBL3095	P33434	0.172563	1 /　6
6,10,14-trimethyl-2-Pentadecanone	Acacb	CHEMBL3108631	E9Q4Z2	0.112042	262 /　0
1-Nonanol	Trpm8	CHEMBL3108632	Q8R4D5	0.166712	1 月 1 日
Tridecanal	Gck	CHEMBL3112387	P52792	0.031227	11 /　0
6,10,14-trimethyl-2-Pentadecanone	Ces1d	CHEMBL3137293	Q8VCT4	0.174863	10 /　12
2,6,6-trimethyl-1-Cyclohexene-1-carboxaldehyde	Nr3c1	CHEMBL3144	P06537	0.043919	0 /　2
Hexadecanoic acid,15-methyl-, methyl ester	Tyms	CHEMBL3160	P07607	0.100579	11 /　0
Dodecanal	Impdh2	CHEMBL3169	P24547	0.071787	24 /　0

7,9-Di-tert-butyl-1-oxaspiro(4,5)deca-6,9-diene-2,8-dione	Chrm2	CHEMBL3197	Q9ERZ4	0.100579	49 /　0
Cyanidin-3-O-glucoside	Ache	CHEMBL3198	P21836	0.123138	0 /　13
（E,Z)-3,6-Nonadien-1-ol	Ada	CHEMBL3206	P03958	0.023832743	15 /　0
6,10,14-trimethyl-2-Pentadecanone	Oprd1	CHEMBL3222	P32300	0.112042	62 /　0
7,9-Di-tert-butyl-1-oxaspiro(4,5)deca-6,9-diene-2,8-dione	Tnks	CHEMBL3232702	Q6PFX9	0.100579	38 /　0
7,9-Di-tert-butyl-1-oxaspiro(4,5)deca-6,9-diene-2,8-dione	Tnks2	CHEMBL3232703	Q3UES3	0.100579	58 /　0
6,10,14-trimethyl-2-Pentadecanone	Nampt	CHEMBL3259474	Q99KQ4	0.112042	113 /　0
7,9-Di-tert-butyl-1-oxaspiro(4,5)deca-6,9-diene-2,8-dione	Hrh3	CHEMBL3263	P58406	0.100579	40 /　0
4-tert-Butylbenzoic acid	Rarb	CHEMBL3266	P22605	0.08057	0 /　37
Protocatechuic acid	Comt	CHEMBL3286068	O88587	0.043919	0 /　7
Cyanidin-3-O-glucoside	Rps6ka3	CHEMBL3297641	P18654	0.114839	0 /　19
7,9-Di-tert-butyl-1-oxaspiro(4,5)deca-6,9-diene-2,8-dione	Ptpn1	CHEMBL3336	P35821	0.100579	19 /　28
6-methyl-5-Hepten-2-one	Chrna7	CHEMBL3365	P49582	0.023833	1 /　0
4-tert-Butylbenzoic acid	Kmo	CHEMBL3407318	Q91WN4	0.071787	7 /　0
Undecanal	Paox	CHEMBL3408	Q8C0L6	0.116739	0 /　1
Hexadecanoic acid,15-methyl-, methyl ester	Alox5ap	CHEMBL3414408	P30355	0.100579	416 /　0
5,9,13-trimehtyl-4,8,12-Tetradecatrienal	Avpr1a	CHEMBL3414410	Q62463	0.053556	22 /　0
7,9-Di-tert-butyl-1-oxaspiro(4,5)deca-6,9-diene-2,8-dione	Scn9a	CHEMBL3414411	Q62205	0.100579	35 /　0
Cyanidin-3-O-glucoside	Cd38	CHEMBL3425388	P56528	0.999915	1 /　2
Dodecanal	Drd2	CHEMBL3427	P61168	0.071787	94 /　0
Hexadecanoic acid,15-methyl-, methyl ester	Ghsr	CHEMBL3428	Q99P50	0.100579	19 /　0
2,6,6-trimethyl-1-Cyclohexene-1-carboxaldehyde	Faah	CHEMBL3455	O08914	0.072716	0 /　14
2,6,6-trimethyl-1-Cyclohexene-1-carboxaldehyde	Nos2	CHEMBL3464	P29477	0.043919	6 月 21 日
2,6,6-trimethyl-1-Cyclohexene-1-carboxaldehyde	Sigmar1	CHEMBL3465	O55242	0.082288	3 月 3 日
Hexadecanoic acid,15-methyl-, methyl ester	Abcb1b	CHEMBL3467	P06795	0.100579	10 /　0
6,10,14-trimethyl-2-Pentadecanone	Mdm2	CHEMBL3600279	P23804	0.112042	260 /　0
7,9-Di-tert-butyl-1-oxaspiro(4,5)deca-6,9-diene-2,8-dione	Egfr	CHEMBL3608	Q01279	0.100579	250 /　0
Chlorogenic acid	Mmp13	CHEMBL3638350	P33435	0.172563	2 /　3
6,10,14-trimethyl-2-Pentadecanone	Ccr5	CHEMBL3676	P51682	0.112042	21 /　0
Dodecanal	Maoa	CHEMBL3681	Q64133	0.071787	87 /　0
Cyanidin-3-O-glucoside	Adora1	CHEMBL3688	Q60612	0.106543	0 /　7
6,10,14-trimethyl-2-Pentadecanone	Chrm1	CHEMBL3733	P12657	0.112042	96 /　0
Formic acid, 2,4,4-trimethylpentyl ester	Parp1	CHEMBL3740	P11103	0.043919	1 /　0
6,10,14-trimethyl-2-Pentadecanone	Npy5r	CHEMBL3802	O70342	0.112042	269 /　0
Hexadecanoic acid,15-methyl-, methyl ester	Raf1	CHEMBL3804748	Q99N57	0.100579	67 /　0
Hexadecanoic acid,15-methyl-, methyl ester	Fgfr4	CHEMBL3839	Q03142	0.100579	3 /　0
6,10,14-trimethyl-2-Pentadecanone	Ccr1	CHEMBL3872	P51675	0.112042	89 /　0
Hexadecanoic acid,15-methyl-, methyl ester	Hsd11b1	CHEMBL3910	P50172	0.100579	278 /　25
6,10,14-trimethyl-2-Pentadecanone	Ptafr	CHEMBL3993	Q62035	0.112042	85 /　0
7,9-Di-tert-butyl-1-oxaspiro(4,5)deca-6,9-diene-2,8-dione	Hdac1	CHEMBL4001	O09106	0.100579	47 /　0
（E,Z)-3,6-Nonadien-1-ol	Cdk1	CHEMBL4084	P11440	0.03397069	19 /　0
Undecanal	Ephx2	CHEMBL4140	P34914	0.125076	117 /　0
4-tert-Butylbenzoic acid	Rarg	CHEMBL4177	P18911	0.08057	0 /　36
6,10,14-trimethyl-2-Pentadecanone	Hsp90aa1	CHEMBL4197	P07901	0.112042	61 /　0

6,10,14-trimethyl-2-Pentadecanone	Ptgs2	CHEMBL4321	Q05769	0.112042	191 / 0
Dodecanal	Hrh1	CHEMBL4322	P70174	0.071787	18 / 0
1-Octen-3-ol	Oprk1	CHEMBL4329	P33534	0.023833	31 / 0
（2Z）-3-pentyl-2,4-Pentadien-1-ol	Rxrg	CHEMBL4402	P28705	0.001	0 / 5
Caffeic acid	Hcar2	CHEMBL4420	Q9EP66	0.08057	0 / 1
Hexadecanal	Soat1	CHEMBL4464	Q61263	0.048953	11 / 0
Vanillic acid	Fyn	CHEMBL4517	P39688	0.125076	3 / 1
Chlorogenic acid	Dhfr	CHEMBL4564	P00375	0.001	2 / 0
Vanillic acid	Polb	CHEMBL4565	Q8K409	0.116739	0 / 1
Chlorogenic acid	Bace1	CHEMBL4593	P56818	0.09724	0 / 3
7,9-Di-tert-butyl-1-oxaspiro(4,5)deca-6,9-diene-2,8-dione	Slc6a4	CHEMBL4642	Q60857	0.100579	45 / 0
3-Isopropylidene-5-methyl-hex-4-en-2-one	Htr7	CHEMBL4764	P32304	0.053518	1 / 0
3-t-Butyl-oct-6-en-1-ol	Gcgr	CHEMBL4773	Q61606	0.071787	35 / 0
3-t-Butyl-oct-6-en-1-ol	Kcna3	CHEMBL4818	P16390	0.071787	13 / 0
7,9-Di-tert-butyl-1-oxaspiro(4,5)deca-6,9-diene-2,8-dione	Taar1	CHEMBL4908	Q923Y8	0.100579	51 / 0
(R)-(-)-(Z)-14-methyl-8-hexadecen-1-ol	Prep	CHEMBL4935	Q9QUR6	0.053556	0 / 1
2,6,6-trimethyl-1-Cyclohexene-1-carboxaldehyde	Prkch	CHEMBL4992	P23298	0.053518	0 / 1
7,9-Di-tert-butyl-1-oxaspiro(4,5)deca-6,9-diene-2,8-dione	Scn10a	CHEMBL5158	Q6QIY3	0.100579	18 / 0
6,10,14-trimethyl-2-Pentadecanone	P2rx7	CHEMBL5183	Q9Z1M0	0.112042	204 / 0
7,9-Di-tert-butyl-1-oxaspiro(4,5)deca-6,9-diene-2,8-dione	Ctsb	CHEMBL5187	P10605	0.100579	109 / 0
Pentadecanal	Cxcr3	CHEMBL5200	O88410	0.060425	8 / 0
Undecanal	Alox5	CHEMBL5211	P48999	0.116739	23 / 0
7,9-Di-tert-butyl-1-oxaspiro(4,5)deca-6,9-diene-2,8-dione	Jak3	CHEMBL5250	Q62137	0.100579	127 / 0
(R)-(-)-(Z)-14-methyl-8-hexadecen-1-ol	Gpr119	CHEMBL5263	Q7TQP3	0.053556	1 / 0
Hexadecanoic acid,15-methyl-, methyl ester	Slc22a20	CHEMBL5269	Q80UJ1	0.100579	0 / 5
3-t-Butyl-oct-6-en-1-ol	Scd1	CHEMBL5353	P13516	0.071787	0 / 1
Vanillic acid	Fbp1	CHEMBL5360	Q9QXD6	0.125076	13 / 0
trans-2-Undecen-1-ol	Cnr2	CHEMBL5373	P47936	0.116739	21 / 1
Cyclohexanol	Shh	CHEMBL5387	Q62226	0.074316	0 / 10
Caffeic acid	Stat3	CHEMBL5402	P42227	0.071787	0 / 1
Hexadecanoic acid,15-methyl-, methyl ester	Ffar1	CHEMBL5411	Q76JU9	0.100579	5 / 2
6,10,14-trimethyl-2-Pentadecanone	Ccr2	CHEMBL5412	P51683	0.112042	5 / 0
2,6,6-trimethyl-1-Cyclohexene-1-carboxaldehyde	Mapk3	CHEMBL5510	Q63844	0.053518	0 / 1
4-tert-Butylbenzoic acid	Pla2g2d	CHEMBL5537	Q9WVF6	0.08057	0 / 1
7,9-Di-tert-butyl-1-oxaspiro(4,5)deca-6,9-diene-2,8-dione	Csf1r	CHEMBL5570	P09581	0.100579	46 / 0
7,9-Di-tert-butyl-1-oxaspiro(4,5)deca-6,9-diene-2,8-dione	Ctsg	CHEMBL5622	P28293	0.100579	5 / 0
Hexadecanoic acid,15-methyl-, methyl ester	Slc22a6	CHEMBL5653	Q8VC69	0.100579	0 / 2
7,9-Di-tert-butyl-1-oxaspiro(4,5)deca-6,9-diene-2,8-dione	Hrh4	CHEMBL5657	Q91ZY2	0.100579	25 / 0
Hexadecanoic acid,15-methyl-, methyl ester	Mcl1	CHEMBL5768	P97287	0.100579	32 / 0
7,9-Di-tert-butyl-1-oxaspiro(4,5)deca-6,9-diene-2,8-dione	Fap	CHEMBL5769	P97321	0.100579	49 / 0
Protocatechuic acid	Alk	CHEMBL5771	P97793	0.043919	0 / 3
6,10,14-trimethyl-2-Pentadecanone	Mgll	CHEMBL5774	O35678	0.112042	48 / 0
7,9-Di-tert-butyl-1-oxaspiro(4,5)deca-6,9-diene-2,8-dione	Akt1	CHEMBL5859	P31750	0.100579	14 / 0
Vanillic acid	Ca15	CHEMBL5973	Q99N23	0.125076	4 / 2

(R)-(-)-(Z)-14-methyl-8-hexadecen-1-ol	Smo	CHEMBL6080	P56726	0.053556	12 / 0

Table S3. Summary of AH-Gene Associations. In DisGeNET database, 656 AH-related genes were obtained.

Disease	Gene	UniProt	DSI_g	DPI_g	Score_gda	First_Ref	Last_Ref
Hepatitis	IFNG	P01579	0.288	0.962	0.4	1986	2020
Hepatitis	FASLG	P48023	0.43	0.885	0.4	2009	2009
Hepatitis	ALB	P02768	0.317	0.962	0.36	2006	2018
Hepatitis	HMOX1	P09601	0.381	0.923	0.33	2003	2019
Hepatitis	CAT	P04040	0.359	0.962	0.32	1991	2017
Hepatitis	PARP1	P09874	0.389	0.923	0.32	2010	2017
Hepatitis	ATP7B	P35670	0.529	0.654	0.31	2003	2007
Hepatitis	SPARC	P09486	0.445	0.846	0.3	2013	2013
Hepatitis	ENPP2	Q13822	0.582	0.769	0.3	2007	2007
Hepatitis	F2R	P25116	0.444	0.846	0.3	2011	2011
Hepatitis	TF	P02787	0.527	0.846	0.3	2011	2011
Hepatitis	GZMB	P10144	0.453	0.808	0.3	2009	2009
Hepatitis	KEAP1	Q14145	0.475	0.808	0.3	2010	2010
Hepatitis	GNMT	Q14749	0.626	0.462	0.3	2010	2010
Hepatitis	INS	P01308	0.445	0.923	0.3	2002	2002
Hepatitis	FOXP3	Q9BZS1	0.368	0.846	0.14	2011	2019
Hepatitis	SERPINA1	P01009	0.41	0.923	0.14	2006	2011
Hepatitis	FAS	P25445	0.372	0.923	0.12	2000	2003
Hepatitis	STAT1	P42224	0.399	0.885	0.11	2011	2011
Hepatitis	CLEC7A	Q9BXN2	0.553	0.769	0.11	2017	2017
Hepatitis	TCF4	P15884	0.464	0.846	0.1		
Hepatitis	CASC15		0.603	0.577	0.1	2014	2014
Hepatitis	GABRG3	Q99928	0.711	0.423	0.1	2014	2014
Hepatitis	TCF3	P15923	0.476	0.846	0.1		
Hepatitis	C1S	P09871	0.705	0.462	0.1		
Hepatitis	BTK	Q06187	0.464	0.808	0.1		
Hepatitis	TGFB1	P01137	0.287	0.962	0.1	1995	2019
Hepatitis	IRF5	Q13568	0.489	0.731	0.1		
Hepatitis	TNF	P01375	0.231	0.962	0.1	1999	2020
Hepatitis	TP53	P04637	0.236	0.962	0.1	1992	2019
Hepatitis	NLRP3	Q96P20	0.361	0.962	0.1	2013	2019
Hepatitis	IL17RA	Q96F46	0.529	0.808	0.1		
Hepatitis	SPIB	Q01892	0.628	0.538	0.1		
Hepatitis	POU2AF1	Q16633	0.608	0.615	0.1		
Hepatitis	GLIS3	Q8NEA6	0.59	0.654	0.1		
Hepatitis	LRRC8A	Q8IWT6	0.644	0.692	0.1		
Hepatitis	TTC7A	Q9ULT0	0.641	0.577	0.1		
Hepatitis	AFP	P02771	0.429	0.885	0.1	2004	2019
Hepatitis	ATP7A	Q04656	0.494	0.769	0.1		
Hepatitis	PIK3R1	P27986	0.477	0.885	0.1		

Hepatitis	PRKCA	Q05655	0.48	0.846	0.1		
Hepatitis	PGM1	P36871	0.628	0.577	0.1		
Hepatitis	TNFSF15	O95150	0.531	0.692	0.1		
Hepatitis	SKIV2L	Q15477	0.615	0.654	0.1		
Hepatitis	MST1	P26927	0.465	0.885	0.1		
Hepatitis	TNPO3	Q9Y5L0	0.663	0.577	0.1		
Hepatitis	SHPK	Q9UHJ6	0.743	0.346	0.1		
Hepatitis	IL22	Q9GZX6	0.393	0.885	0.1	2004	2019
Hepatitis	CYP7A1	P22680	0.576	0.615	0.1		
Hepatitis	TPP2	P29144	0.729	0.462	0.1		
Hepatitis	RASGRP1	O95267	0.572	0.731	0.1		
Hepatitis	ITCH	Q96J02	0.556	0.769	0.1		
Hepatitis	IL33	O95760	0.409	0.885	0.1	2012	2019
Hepatitis	SYT16	Q17RD7			0.1	2014	2014
Hepatitis	CASP10	Q92851	0.528	0.692	0.1		
Hepatitis	IGLL1	P15814	0.678	0.538	0.1		
Hepatitis	IL17RC	Q8NAC3	0.633	0.615	0.1		
Hepatitis	CYP7B1	O75881	0.563	0.692	0.1		
Hepatitis	BLNK	Q8WV28	0.603	0.731	0.1		
Hepatitis	CD247	P20963	0.539	0.731	0.1		
Hepatitis	NAV2	Q8IVL1	0.722	0.385	0.1	2014	2014
Hepatitis	CD3E	P07766	0.615	0.615	0.1		
Hepatitis	GUSB	P08236	0.53	0.846	0.1		
Hepatitis	CD3D	P04234	0.626	0.577	0.1		
Hepatitis	SLC25A15	Q9Y619	0.711	0.346	0.1		
Hepatitis	TBX19	O60806	0.695	0.462	0.1		
Hepatitis	IL1B	P01584	0.276	0.962	0.1	2007	2019
Hepatitis	GPT	P24298	0.403	0.923	0.1	1991	2019
Hepatitis	IL17F	Q96PD4	0.48	0.808	0.1		
Hepatitis	IL17A	Q16552	0.324	0.923	0.1	2012	2019
Hepatitis	IL12RB1	P42701	0.555	0.731	0.1		
Hepatitis	IL12A	P29459	0.491	0.808	0.1		
Hepatitis	IGHM	P01871	0.644	0.654	0.1		
Hepatitis	TLR4	O00206	0.321	0.962	0.1	2012	2019
Hepatitis	CD79B	P40259	0.576	0.577	0.1		
Hepatitis	IL6	P05231	0.248	0.962	0.1	2006	2018
Hepatitis	TRAF3IP2	O43734	0.585	0.692	0.1		
Hepatitis	GPR35	Q9HC97	0.587	0.654	0.1		
Hepatitis	CD79A	P11912	0.536	0.731	0.1		
Hepatitis	XIAP	P98170	0.447	0.808	0.1		
Hepatitis	MMEL1	Q495T6	0.606	0.692	0.1		
Hepatitis	SLC17A5	Q9NRA2	0.491	0.808	0.09	2015	2019
Hepatitis	PTGS2	P35354	0.314	0.962	0.08	2007	2019
Hepatitis	MIR122		0.468	0.808	0.08	2012	2018

Hepatitis	CYP2E1	P05181	0.459	0.885	0.07	2003	2018
Hepatitis	IL4	P05112	0.332	0.962	0.07	1995	2019
Hepatitis	IL10	P22301	0.281	0.923	0.07	2001	2020
Hepatitis	IFNL3	Q8IZI9	0.506	0.808	0.07	2010	2017
Hepatitis	CXCL10	P02778	0.378	0.885	0.07	2000	2019
Hepatitis	IL18	Q14116	0.365	0.923	0.06	2002	2019
Hepatitis	CCL4	P13236;Q8NHW4	0.466	0.923	0.06	2007	2019
Hepatitis	IFNA1	P01562	0.371	0.923	0.06	1988	2011
Hepatitis	HLA-DRB1	P01911	0.333	0.923	0.06	1998	2009
Hepatitis	HMGB1	P09429	0.368	0.923	0.05	2014	2019
Hepatitis	KLRK1	P26718	0.467	0.769	0.05	2007	2018
Hepatitis	GOLM1	Q8NBJ4	0.56	0.692	0.05	2000	2019
Hepatitis	CD274	Q9NZQ7	0.324	0.923	0.05	2012	2018
Hepatitis	IFNA13	P01562	0.374	0.923	0.05	1988	2011
Hepatitis	NOS2	P35228	0.364	0.923	0.05	1997	2017
Hepatitis	IL1A	P01583	0.333	0.962	0.05	2013	2019
Hepatitis	COX2	P00403	0.352	0.962	0.05	2008	2019
Hepatitis	SPP1	P10451	0.353	0.885	0.05	2004	2012
Hepatitis	VIPR1	P32241	0.573	0.654	0.05	1992	2015
Hepatitis	BCL2	P10415	0.291	0.885	0.05	1999	2019
Hepatitis	SOCS3	O14543	0.434	0.808	0.05	2006	2018
Hepatitis	TLR2	O60603	0.361	0.923	0.05	2012	2018
Hepatitis	PDLIM5	Q96HC4	0.525	0.808	0.05	2010	2016
Hepatitis	MTCO2P12		0.368	0.962	0.04	2008	2019
Hepatitis	FBL	P22087	0.572	0.654	0.04	2017	2019
Hepatitis	TNFSF10	P50591	0.413	0.885	0.04	2008	2017
Hepatitis	CCL2	P13500	0.321	0.962	0.04	2017	2018
Hepatitis	MYD88	Q99836	0.414	0.923	0.04	2012	2019
Hepatitis	GSTM1	P09488	0.38	0.923	0.04	1987	2010
Hepatitis	RIPK1	Q13546	0.479	0.769	0.04	2012	2017
Hepatitis	IL1RN	P18510	0.373	0.923	0.04	2012	2019
Hepatitis	CASP1	P29466	0.413	0.885	0.04	2017	2018
Hepatitis	CXCL2	P19875	0.513	0.885	0.04	2011	2018
Hepatitis	NAT2	P11245	0.451	0.885	0.04	1997	2011
Hepatitis	CXCL8	P10145	0.31	0.962	0.04	2005	2017
Hepatitis	LEP	P41159	0.349	0.846	0.04	2010	2018
Hepatitis	CTNNB1	P35222	0.303	0.885	0.04	2000	2019
Hepatitis	RBM45	Q8IUH3	0.383	0.846	0.04	1998	2008
Hepatitis	MIR223		0.433	0.808	0.04	2015	2019
Hepatitis	RIPK3	Q9Y572	0.497	0.885	0.03	2012	2019
Hepatitis	GGT1	P19440	0.496	0.885	0.03	2006	2019
Hepatitis	GCG	P01275	0.431	0.885	0.03	2014	2018
Hepatitis	LGALS3	P17931	0.392	0.846	0.03	2012	2019
Hepatitis	IL2	P60568	0.336	0.885	0.03	1995	2007

Hepatitis	LGALS9	O00182	0.496	0.769	0.03	2012	2017
Hepatitis	ABCC2	Q92887	0.483	0.808	0.03	2007	2020
Hepatitis	IL27	Q8NEV9	0.457	0.808	0.03	2015	2018
Hepatitis	SMAD2	Q15796	0.456	0.808	0.03	2018	2019
Hepatitis	SIRT1	Q96EB6	0.378	0.885	0.03	2013	2017
Hepatitis	STAT3	P40763	0.32	0.923	0.03	2002	2018
Hepatitis	EHMT1	Q9H9B1	0.46	0.808	0.03	2018	2018
Hepatitis	IL5	P05113	0.437	0.846	0.03	2007	2019
Hepatitis	LGALS1	P09382	0.435	0.846	0.03	2014	2020
Hepatitis	IL17D	Q8TAD2	0.464	0.808	0.03	2015	2018
Hepatitis	TLR9	Q9NR96	0.411	0.885	0.03	2018	2019
Hepatitis	HGF	P14210	0.374	0.885	0.03	1991	2019
Hepatitis	GLP1R	P43220	0.471	0.846	0.03	2014	2018
Hepatitis	HFE	Q30201	0.436	0.846	0.03	2001	2005
Hepatitis	CCN2	P29279	0.399	0.846	0.03	2012	2016
Hepatitis	IFNA2	P01563	0.442	0.885	0.03	2001	2017
Hepatitis	KLRC4-KLRK1	P26718	0.469	0.769	0.03	2013	2018
Hepatitis	HIF1A	Q16665	0.327	0.923	0.03	2008	2019
Hepatitis	NFE2L2	Q16236	0.357	0.885	0.03	2017	2018
Hepatitis	AGT	P01019	0.367	0.923	0.03	1996	2018
Hepatitis	PAEP	P09466	0.43	0.846	0.03	2005	2017
Hepatitis	ESRRB	O95718	0.565	0.846	0.03	2017	2018
Hepatitis	GSTT1	P30711	0.393	0.923	0.03	2007	2013
Hepatitis	TNFRSF9	Q07011	0.502	0.769	0.02	2010	2018
Hepatitis	VDR	P11473	0.352	0.885	0.02	2019	2019
Hepatitis	CLOCK	O15516	0.463	0.885	0.02	2013	2018
Hepatitis	ICAM1	P05362	0.364	0.962	0.02	1992	1995
Hepatitis	CD40	P25942	0.396	0.846	0.02	2018	2019
Hepatitis	IL13	P35225	0.386	0.846	0.02	2017	2019
Hepatitis	HLA-G	P17693	0.461	0.769	0.02	2014	2015
Hepatitis	KRT18	P05783	0.53	0.692	0.02	2007	2017
Hepatitis	GGTLC4P		0.513	0.846	0.02	2018	2019
Hepatitis	TXN	P10599	0.447	0.885	0.02	2003	2006
Hepatitis	CCL4L2	P13236;Q8NHW4	0.535	0.885	0.02	2007	2011
Hepatitis	CASP8	Q14790	0.404	0.923	0.02	2012	2018
Hepatitis	NR0B2	Q15466	0.474	0.808	0.02	2018	2018
Hepatitis	GPT2	Q8TD30	0.65	0.577	0.02	2006	2018
Hepatitis	SPAG9	O60271	0.566	0.769	0.02	2017	2017
Hepatitis	TNFAIP8L2	Q6P589	0.575	0.615	0.02	2011	2017
Hepatitis	APOE	P02649	0.338	0.962	0.02	2002	2018
Hepatitis	IFNB1	P01574	0.421	0.846	0.02	2011	2019
Hepatitis	GRAP2	O75791	0.394	0.923	0.02	2011	2019
Hepatitis	MBOAT7	Q96N66	0.612	0.5	0.02	2016	2019
Hepatitis	MANF	P55145	0.621	0.654	0.02	2018	2019

Hepatitis	PTGES	O14684	0.54	0.769	0.02	2007	2018
Hepatitis	CCR2	P41597	0.418	0.846	0.02	2017	2018
Hepatitis	AHI1	Q8N157	0.513	0.846	0.02	1999	2003
Hepatitis	NCKIPSD	Q9NZQ3	0.612	0.577	0.02	1999	2003
Hepatitis	TLR7	Q9NYK1	0.464	0.885	0.02	2013	2019
Hepatitis	RIT2	Q99578	0.647	0.538	0.02	1993	1994
Hepatitis	PCSK1	P29120	0.548	0.615	0.02	2012	2015
Hepatitis	RNASEL	Q05823	0.579	0.692	0.02	2012	2013
Hepatitis	OPN1SW	P03999	0.519	0.769	0.02	2011	2015
Hepatitis	NOX4	Q9NPH5	0.471	0.885	0.02	2015	2015
Hepatitis	CCL16	O15467	0.686	0.5	0.02	1997	1999
Hepatitis	P2RX7	Q99572	0.45	0.808	0.02	2017	2019
Hepatitis	RELA	Q04206	0.406	0.885	0.02	2005	2007
Hepatitis	PECAM1	P16284	0.426	0.846	0.02	2000	2015
Hepatitis	UGT1A1	P22309	0.464	0.769	0.02	2018	2018
Hepatitis	PPARA	Q07869	0.432	0.885	0.02	2014	2017
Hepatitis	MAPK8	P45983	0.397	0.885	0.02	2016	2019
Hepatitis	PON1	P27169	0.409	0.885	0.02	2011	2017
Hepatitis	MYDGF	Q969H8	0.451	0.846	0.02	2015	2018
Hepatitis	RETN	Q9HD89	0.454	0.808	0.02	2012	2014
Hepatitis	KLHL1	Q9NR64	0.569	0.654	0.02	2007	2020
Hepatitis	IL21	Q9HBE4	0.441	0.769	0.02	2011	2018
Hepatitis	MAPK1	P28482	0.33	0.923	0.02	2011	2019
Hepatitis	NOTCH1	P46531	0.369	0.885	0.02	2017	2018
Hepatitis	CXCL11	O14625	0.519	0.808	0.02	2015	2019
Hepatitis	NFKB1	P19838	0.396	0.923	0.02	2017	2018
Hepatitis	LTBR	P36941	0.592	0.692	0.02	2007	2017
Hepatitis	EPCAM	P16422	0.451	0.885	0.02	2018	2019
Hepatitis	CCL4L1	P13236;Q8NHW4	0.534	0.885	0.02	2007	2011
Hepatitis	ADAM17	P78536	0.453	0.808	0.02	2014	2016
Hepatitis	GSTK1	Q9Y2Q3	0.412	0.885	0.02	2010	2017
Hepatitis	JUN	P05412	0.442	0.885	0.02	2007	2013
Hepatitis	ISG20	Q96AZ6	0.414	0.846	0.02	2018	2019
Hepatitis	TLR3	O15455	0.427	0.885	0.02	2016	2018
Hepatitis	GGTLC3	B5MD39	0.513	0.846	0.02	2018	2019
Hepatitis	MIR146A		0.398	0.885	0.02	2017	2018
Hepatitis	MIR155		0.384	0.885	0.02	2013	2016
Hepatitis	S100A4	P26447	0.448	0.769	0.02	2018	2019
Hepatitis	MYC	P01106	0.344	0.923	0.02	1992	2009
Hepatitis	CXCL12	P48061	0.379	0.846	0.02	2003	2019
Hepatitis	GGTLC5P		0.513	0.846	0.02	2018	2019
Hepatitis	MEFV	O15553	0.44	0.885	0.02	2018	2018
Hepatitis	AIMP2	Q13155	0.393	0.923	0.02	2011	2019
Hepatitis	SMN1	Q16637	0.454	0.769	0.02	2019	2019

Hepatitis	SMN2	Q16637	0.459	0.769	0.02	2019	2019
Hepatitis	MIR29A		0.459	0.808	0.02	2014	2019
Hepatitis	GGT2	P36268	0.513	0.846	0.02	2018	2019
Hepatitis	AHSA1	O95433	0.396	0.923	0.02	2011	2019
Hepatitis	CDKN2A	P42771;Q8N726	0.3	0.885	0.02	2004	2004
Hepatitis	CRK	P46108	0.394	0.923	0.02	2011	2019
Hepatitis	IL18BP	O95998	0.603	0.731	0.02	2013	2019
Hepatitis	ABCB6	Q9NP58	0.431	0.885	0.02	1997	2013
Hepatitis	GABPA	Q06546	0.379	0.885	0.02	2018	2018
Hepatitis	GSR	P00390	0.494	0.846	0.02	2017	2017
Hepatitis	FCGR2B	P31994	0.522	0.846	0.02	2018	2019
Hepatitis	FGL2	Q14314	0.621	0.615	0.02	2005	2017
Hepatitis	CXCR3	P49682	0.436	0.808	0.02	2008	2015
Hepatitis	IFNL1	Q8IU54	0.599	0.654	0.02	2006	2011
Hepatitis	CREB1	P16220	0.463	0.885	0.02	2018	2018
Hepatitis	MAPK14	Q16539	0.379	0.923	0.02	2011	2019
Hepatitis	SLCO6A1	Q86UG4	0.412	0.885	0.02	2010	2017
Hepatitis	EMB	Q6PCB8	0.633	0.615	0.02	1994	1994
Hepatitis	AHR	P35869	0.41	0.923	0.02	2014	2020
Hepatitis	TXNIP	Q9H3M7	0.49	0.731	0.02	2013	2017
Hepatitis	F10	P00742	0.493	0.769	0.02	2005	2018
Hepatitis	EPHX1	P07099	0.5	0.846	0.02	2007	2009
Hepatitis	RNF19A	Q9NV58	0.397	0.923	0.02	2011	2019
Hepatitis	ACE	P12821	0.328	0.923	0.02	2017	2019
Hepatitis	CTLA4	P16410	0.369	0.923	0.02	2014	2017
Hepatitis	SYCE1L	A8MT33	0.57	0.654	0.02	2007	2020
Hepatitis	ZGLP1	P0C6A0	0.512	0.808	0.02	2018	2018
Hepatitis	HLA-A	P04439	0.37	0.846	0.02	1981	2011
Hepatitis	IL34	Q6ZMJ4	0.56	0.769	0.02	2018	2019
Hepatitis	CD24	P25063	0.472	0.769	0.02	2017	2018
Hepatitis	EXT1	Q16394	0.522	0.769	0.02	2001	2003
Hepatitis	POLDIP2	Q9Y2S7	0.396	0.923	0.02	2011	2019
Hepatitis	COL15A1	P39059	0.682	0.577	0.01	2016	2016
Hepatitis	CIDEA	O60543	0.751	0.269	0.01	2019	2019
Hepatitis	VASN	Q6EMK4	0.722	0.269	0.01	2015	2015
Hepatitis	COMP	P49747	0.503	0.808	0.01	2017	2017
Hepatitis	IL17RE	Q8NFR9	0.751	0.385	0.01	2017	2017
Hepatitis	SOX11	P35716	0.494	0.808	0.01	2016	2016
Hepatitis	SON	P18583	0.565	0.769	0.01	2015	2015
Hepatitis	SPINT1	O43278	0.57	0.577	0.01	2001	2001
Hepatitis	NR2C2	P49116	0.534	0.808	0.01	2018	2018
Hepatitis	ACOT7	O00154	0.531	0.769	0.01	2017	2017
Hepatitis	TP73	O15350	0.449	0.808	0.01	1999	1999
Hepatitis	MAP3K8	P41279	0.516	0.846	0.01	2019	2019

Hepatitis	SMPD1	P17405	0.499	0.885	0.01	2017	2017
Hepatitis	H3P16		0.705	0.346	0.01	2005	2005
Hepatitis	ACTG1	P63261	0.477	0.846	0.01	2017	2017
Hepatitis	MGLL	Q99685	0.564	0.731	0.01	2019	2019
Hepatitis	PGLYRP2	Q96PD5	0.805	0.231	0.01	2013	2013
Hepatitis	AKR1D1	P51857	0.711	0.385	0.01	2003	2003
Hepatitis	H3P10		0.35	0.846	0.01	2004	2004
Hepatitis	H3P40		0.506	0.846	0.01	2018	2018
Hepatitis	MAP3K7	O43318	0.477	0.808	0.01	2018	2018
Hepatitis	TAZ	Q16635	0.477	0.808	0.01	2018	2018
Hepatitis	SERPINA7	P05543	0.641	0.615	0.01	1988	1988
Hepatitis	CCR4	P51679	0.51	0.731	0.01	2020	2020
Hepatitis	PLD4	Q96BZ4	0.78	0.5	0.01	2018	2018
Hepatitis	MIR602		0.792	0.231	0.01	2010	2010
Hepatitis	TGFA	P01135	0.432	0.885	0.01	2006	2006
Hepatitis	CLTC	Q00610	0.558	0.769	0.01	2013	2013
Hepatitis	CLU	P10909	0.426	0.885	0.01	2018	2018
Hepatitis	SERPINA3	P01011	0.486	0.846	0.01	2017	2017
Hepatitis	TGFB2	P61812	0.433	0.885	0.01	2015	2015
Hepatitis	BSG	P35613	0.458	0.769	0.01	2019	2019
Hepatitis	TRIM21	P19474	0.485	0.808	0.01	2020	2020
Hepatitis	RO60	P10155	0.61	0.692	0.01	1999	1999
Hepatitis	COL11A2	P13942	0.435	0.846	0.01	2017	2017
Hepatitis	CNR1	P21554	0.446	0.808	0.01	2017	2017
Hepatitis	TIMP2	P16035	0.425	0.846	0.01	2001	2001
Hepatitis	SYT1	P21579	0.412	0.846	0.01	2005	2005
Hepatitis	CMKLR1	Q99788	0.612	0.692	0.01	2013	2013
Hepatitis	TDO2	P48775	0.599	0.654	0.01	2020	2020
Hepatitis	MPPE1	Q53F39	0.736	0.346	0.01	2019	2019
Hepatitis	MIR451A		0.505	0.769	0.01	2019	2019
Hepatitis	RHD	Q02161	0.612	0.654	0.01	2017	2017
Hepatitis	NCAN	O14594	0.67	0.5	0.01	2013	2013
Hepatitis	CSF3	P09919	0.377	0.885	0.01	2018	2018
Hepatitis	BRD2	P25440	0.45	0.808	0.01	2019	2019
Hepatitis	APOBEC3G	Q9HC16	0.653	0.346	0.01	2006	2006
Hepatitis	CSE1L	P55060	0.521	0.808	0.01	2001	2001
Hepatitis	HCC		0.644	0.423	0.01	2018	2018
Hepatitis	BDH1	Q02338	0.729	0.423	0.01	2015	2015
Hepatitis	RRM2	P31350	0.555	0.769	0.01	2010	2010
Hepatitis	SORT1	Q99523	0.573	0.615	0.01	2017	2017
Hepatitis	ACTB	P60709	0.325	0.923	0.01	2013	2013
Hepatitis	RDX	P35241	0.593	0.615	0.01	2020	2020
Hepatitis	RARRES2	Q99969	0.519	0.731	0.01	2013	2013
Hepatitis	MIR499A		0.519	0.846	0.01	2018	2018

Hepatitis	SEMA6A	Q9H2E6	0.486	0.808	0.01	2018	2018
Hepatitis	COMMD1	Q8N668	0.633	0.692	0.01	2011	2011
Hepatitis	PTPN11	Q06124	0.385	0.923	0.01	1992	1992
Hepatitis	HAMP	P81172	0.433	0.846	0.01	2018	2018
Hepatitis	PTPRO	Q16827	0.656	0.423	0.01	2014	2014
Hepatitis	JAM2	P57087	0.736	0.462	0.01	2018	2018
Hepatitis	RAG2	P55895	0.507	0.769	0.01	2019	2019
Hepatitis	BCHE	P06276	0.447	0.923	0.01	2017	2017
Hepatitis	RAP1A	P62834	0.502	0.808	0.01	2016	2016
Hepatitis	WNK1	Q9H4A3	0.416	0.846	0.01	2005	2005
Hepatitis	S100A9	P06702	0.433	0.885	0.01	2018	2018
Hepatitis	IFIH1	Q9BYX4	0.471	0.808	0.01	2015	2015
Hepatitis	POTEM	A6NI47	0.541	0.769	0.01	2017	2017
Hepatitis	SFPQ	P23246	0.593	0.654	0.01	2007	2007
Hepatitis	CPT1B	Q92523	0.653	0.538	0.01	2016	2016
Hepatitis	CTNND1	O60716	0.5	0.808	0.01	2001	2001
Hepatitis	CPT1A	P50416	0.555	0.808	0.01	2016	2016
Hepatitis	GORASP1	Q9BQQ3	0.428	0.846	0.01	2005	2005
Hepatitis	DEPTOR	Q8TB45	0.626	0.654	0.01	2016	2016
Hepatitis	ST6GAL1	P15907	0.569	0.731	0.01	2017	2017
Hepatitis	COX8A	P10176	0.4	0.846	0.01	1997	1997
Hepatitis	NOD2	Q9HC29	0.423	0.923	0.01	2008	2008
Hepatitis	SAMSN1	Q9NSI8	0.716	0.385	0.01	2015	2015
Hepatitis	SELP	P16109	0.46	0.769	0.01	2010	2010
Hepatitis	S100B	P04271	0.383	0.923	0.01	2008	2008
Hepatitis	SARS1	P49591	0.558	0.769	0.01	2008	2008
Hepatitis	SERPINB3	P29508	0.565	0.615	0.01	2012	2012
Hepatitis	SERPINB4	P48594	0.623	0.423	0.01	2012	2012
Hepatitis	CEACAM1	P13688	0.525	0.731	0.01	2004	2004
Hepatitis	CRP	P02741	0.299	0.962	0.01	2010	2010
Hepatitis	ATF2	P15336	0.551	0.808	0.01	2018	2018
Hepatitis	CCL20	P78556	0.474	0.846	0.01	2002	2002
Hepatitis	CPT2	P23786	0.542	0.769	0.01	2016	2016
Hepatitis	ERVK-6		0.521	0.808	0.01	1994	1994
Hepatitis	SLC4A1	P02730	0.522	0.731	0.01	2013	2013
Hepatitis	TRAF6	Q9Y4K3	0.472	0.808	0.01	2013	2013
Hepatitis	TSLP	Q969D9	0.486	0.808	0.01	2007	2007
Hepatitis	LOC102724197		0.559	0.731	0.01	2019	2019
Hepatitis	ARTN	Q5T4W7	0.48	0.846	0.01	2019	2019
Hepatitis	CDKN1A	P38936	0.403	0.769	0.01	2005	2005
Hepatitis	NAMPT	P43490	0.502	0.808	0.01	2015	2015
Hepatitis	CD1D	P15813	0.532	0.769	0.01	2004	2004
Hepatitis	P2RX6	O15547	0.599	0.769	0.01	2017	2017
Hepatitis	ERVK-20		0.533	0.692	0.01	1994	1994

Hepatitis	MYOM2	P54296	0.57	0.692	0.01	1994	1994
Hepatitis	TIMD4	Q96H15	0.644	0.654	0.01	2019	2019
Hepatitis	DCLK1	O15075	0.545	0.615	0.01	2015	2015
Hepatitis	GGTLC1	Q9BX51	0.559	0.731	0.01	2019	2019
Hepatitis	SLAMF9	Q96A28	0.861	0.192	0.01	2020	2020
Hepatitis	CCNE1	P24864	0.538	0.731	0.01	2018	2018
Hepatitis	SOCS1	O15524	0.445	0.808	0.01	2004	2004
Hepatitis	TLR6	Q9Y2C9	0.556	0.769	0.01	2016	2016
Hepatitis	CDKN2B	P42772	0.417	0.769	0.01	2009	2009
Hepatitis	FADD	Q13158	0.537	0.846	0.01	2006	2006
Hepatitis	PROM1	O43490	0.41	0.846	0.01	2015	2015
Hepatitis	NR1I2	O75469	0.418	0.846	0.01	2010	2010
Hepatitis	ARHGEF7	Q14155	0.486	0.846	0.01	2018	2018
Hepatitis	SPHK1	Q9NYA1	0.48	0.808	0.01	2019	2019
Hepatitis	SQSTM1	Q13501	0.428	0.885	0.01	2018	2018
Hepatitis	DDX18	Q9NVP1	0.805	0.192	0.01	2013	2013
Hepatitis	ADAR	P55265	0.501	0.808	0.01	2017	2017
Hepatitis	CD5L	O43866	0.593	0.846	0.01	2019	2019
Hepatitis	IL32	P24001	0.495	0.731	0.01	2012	2012
Hepatitis	PARP2	Q9UGN5	0.663	0.538	0.01	2017	2017
Hepatitis	BCAR1	P56945	0.518	0.846	0.01	2001	2001
Hepatitis	CD44	P16070	0.363	0.962	0.01	2017	2017
Hepatitis	CD58	P19256	0.584	0.769	0.01	2005	2005
Hepatitis	CD59	P13987	0.469	0.808	0.01	2013	2013
Hepatitis	CD68	P34810	0.408	0.808	0.01	2018	2018
Hepatitis	TEC		0.659	0.577	0.01	2013	2013
Hepatitis	CD81	P60033	0.555	0.846	0.01	2001	2001
Hepatitis	RIPOR2	Q9Y4F9	0.792	0.269	0.01	2017	2017
Hepatitis	ADA	P00813	0.44	0.885	0.01	2017	2017
Hepatitis	CDK1	P06493	0.482	0.808	0.01	2019	2019
Hepatitis	CXCL14	O95715	0.554	0.731	0.01	2019	2019
Hepatitis	MICA	Q29983	0.473	0.846	0.01	2016	2016
Hepatitis	MSC	O60682	0.475	0.846	0.01	2014	2014
Hepatitis	CD14	P08571	0.392	0.885	0.01	2017	2017
Hepatitis	MS4A1	P11836	0.411	0.846	0.01	2019	2019
Hepatitis	TCEAL1	Q15170	0.7	0.346	0.01	2005	2005
Hepatitis	ADIPOQ	Q15848	0.376	0.885	0.01	2015	2015
Hepatitis	P2RX5-TAX1BP3		0.588	0.769	0.01	2017	2017
Hepatitis	CD80	P33681	0.475	0.769	0.01	2019	2019
Hepatitis	FADS2	O95864	0.575	0.692	0.01	2017	2017
Hepatitis	TMX2-CTNND1		0.538	0.769	0.01	2001	2001
Hepatitis	FHL5	Q5TD97	0.53	0.769	0.01	2017	2017
Hepatitis	GDF15	Q99988	0.429	0.808	0.01	2017	2017
Hepatitis	SFRP5	Q5T4F7	0.559	0.692	0.01	2017	2017

Hepatitis	ACTG2	P63267	0.505	0.808	0.01	2017	2017
Hepatitis	VCAM1	P19320	0.422	0.808	0.01	2005	2005
Hepatitis	ERVK-32		0.522	0.692	0.01	1994	1994
Hepatitis	VEGFA	P15692	0.266	0.923	0.01	2017	2017
Hepatitis	VIM	P08670	0.372	0.846	0.01	2019	2019
Hepatitis	VTN	P04004	0.509	0.769	0.01	2008	2008
Hepatitis	XRCC1	P18887	0.421	0.923	0.01	2009	2009
Hepatitis	ALDH1L1	O75891	0.663	0.615	0.01	2017	2017
Hepatitis	FTO	Q9C0B1	0.486	0.885	0.01	2018	2018
Hepatitis	DHX58	Q96C10	0.705	0.308	0.01	2019	2019
Hepatitis	CETP	P11597	0.512	0.731	0.01	2018	2018
Hepatitis	TNFSF13B	Q9Y275	0.46	0.731	0.01	2017	2017
Hepatitis	UROD	P06132	0.521	0.769	0.01	1999	1999
Hepatitis	USP4	Q13107	0.686	0.308	0.01	2019	2019
Hepatitis	VSIG4	Q9Y279	0.628	0.615	0.01	2018	2018
Hepatitis	POTEF	A5A3E0	0.481	0.885	0.01	2013	2013
Hepatitis	ESM1	Q9NQ30	0.595	0.654	0.01	2011	2011
Hepatitis	HULC		0.606	0.538	0.01	2017	2017
Hepatitis	LOC110806263		0.464	0.731	0.01	2017	2017
Hepatitis	TNFRSF4	P43489	0.547	0.692	0.01	2017	2017
Hepatitis	STON1	Q9Y6Q2	0.729	0.308	0.01	2018	2018
Hepatitis	GTF2A1L	Q9UNN4	0.678	0.385	0.01	2018	2018
Hepatitis	TYRO3	Q06418	0.559	0.769	0.01	2020	2020
Hepatitis	UBE2D1	P51668	0.7	0.385	0.01	2018	2018
Hepatitis	UMOD	P07911	0.52	0.846	0.01	2018	2018
Hepatitis	NELFE	P18615	0.686	0.5	0.01	2012	2012
Hepatitis	TFEB	P19484	0.527	0.731	0.01	2018	2018
Hepatitis	CASP3	P42574	0.351	0.923	0.01	2012	2012
Hepatitis	JAM3	Q9BX67	0.619	0.654	0.01	2018	2018
Hepatitis	IVNS1ABP	Q9Y6Y0	0.608	0.769	0.01	1992	1992
Hepatitis	SLCO1B1	Q9Y6L6	0.538	0.731	0.01	2012	2012
Hepatitis	ARFGEF1	Q9Y6D6	0.674	0.423	0.01	2019	2019
Hepatitis	ATG7	O95352	0.505	0.769	0.01	2016	2016
Hepatitis	SMARCA5	O60264	0.67	0.5	0.01	2019	2019
Hepatitis	TRIM55	Q9BYV6	0.821	0.154	0.01	2018	2018
Hepatitis	TRIM63	Q969Q1	0.617	0.538	0.01	2018	2018
Hepatitis	MERTK	Q12866	0.531	0.692	0.01	2016	2016
Hepatitis	YAP1	P46937	0.432	0.808	0.01	2019	2019
Hepatitis	ARHGAP24	Q8N264	0.456	0.808	0.01	1999	1999
Hepatitis	SYMPK	Q92797	0.7	0.231	0.01	2019	2019
Hepatitis	BIRC7	Q96CA5	0.621	0.5	0.01	2013	2013
Hepatitis	CXCR6	O00574	0.448	0.885	0.01	2019	2019
Hepatitis	MARCKS	P29966	0.559	0.769	0.01	2005	2005
Hepatitis	POSTN	Q15063	0.428	0.885	0.01	2015	2015

Hepatitis	RNF34	Q969K3	0.61	0.577	0.01	2019	2019
Hepatitis	HKDC1	Q2TB90	0.743	0.269	0.01	2019	2019
Hepatitis	WDR26	Q9H7D7	0.606	0.769	0.01	2017	2017
Hepatitis	PNPLA3	Q9NST1	0.556	0.692	0.01	2019	2019
Hepatitis	LPAL2	Q16609	0.626	0.731	0.01	2011	2011
Hepatitis	CALR	P27797	0.413	0.923	0.01	2020	2020
Hepatitis	CDR3		0.472	0.808	0.01	2013	2013
Hepatitis	CDSN	Q15517	0.585	0.731	0.01	2008	2008
Hepatitis	BBC3	Q96PG8;Q9BXH1	0.517	0.769	0.01	2010	2010
Hepatitis	KLRB1	Q12918	0.601	0.615	0.01	2004	2004
Hepatitis	KRT7	P08729	0.463	0.808	0.01	2019	2019
Hepatitis	CHKB-CPT1B		0.769	0.385	0.01	2016	2016
Hepatitis	GCKR	Q14397	0.604	0.615	0.01	2016	2016
Hepatitis	LBP	P18428	0.525	0.808	0.01	2012	2012
Hepatitis	LCN2	P80188	0.405	0.885	0.01	2011	2011
Hepatitis	LDLR	P01130	0.449	0.885	0.01	1996	1996
Hepatitis	COPD		0.456	0.885	0.01	2019	2019
Hepatitis	LEPR	P48357	0.433	0.808	0.01	2010	2010
Hepatitis	SETBP1	Q9Y6X0	0.503	0.808	0.01	1995	1995
Hepatitis	TIPARP	Q7Z3E1	0.711	0.346	0.01	2020	2020
Hepatitis	KIR3DL2	P43630	0.543	0.769	0.01	2016	2016
Hepatitis	KIR2DS1	Q14954	0.537	0.769	0.01	2016	2016
Hepatitis	LAMP3	Q9UQV4	0.56	0.692	0.01	2017	2017
Hepatitis	NSG1	P42857	0.691	0.538	0.01	2005	2005
Hepatitis	INSR	P06213	0.432	0.846	0.01	2017	2017
Hepatitis	SIGLEC7	Q9Y286	0.535	0.846	0.01	2019	2019
Hepatitis	IRS1	P35568	0.488	0.769	0.01	2005	2005
Hepatitis	GJA1	P17302	0.393	0.885	0.01	2018	2018
Hepatitis	ITGAM	P11215	0.443	0.808	0.01	2018	2018
Hepatitis	B4GALT1	P15291	0.674	0.538	0.01	2008	2008
Hepatitis	JUNB	P17275	0.467	0.769	0.01	2013	2013
Hepatitis	AMELY	Q99218	0.743	0.269	0.01	2010	2010
Hepatitis	MALAT1		0.435	0.846	0.01	2017	2017
Hepatitis	LGALS3BP	Q08380	0.546	0.769	0.01	2017	2017
Hepatitis	SGMS1	Q86VZ5	0.674	0.577	0.01	2019	2019
Hepatitis	LSAMP	Q13449	0.558	0.654	0.01	2017	2017
Hepatitis	IPMK	Q8NFU5	0.751	0.423	0.01	2019	2019
Hepatitis	FUT8	Q9BYC5	0.603	0.692	0.01	1999	1999
Hepatitis	MIR29B1		0.485	0.846	0.01	2014	2014
Hepatitis	MIR29B2		0.484	0.885	0.01	2014	2014
Hepatitis	MIR29C		0.496	0.808	0.01	2014	2014
Hepatitis	MIR96		0.536	0.769	0.01	2019	2019
Hepatitis	MIR98		0.565	0.731	0.01	2017	2017
Hepatitis	SMUG1	Q53HV7	0.322	0.923	0.01	2019	2019

Hepatitis	PIK3R5	Q8WYR1	0.7	0.5	0.01	2019	2019
Hepatitis	ARC	Q7LC44	0.573	0.769	0.01	2012	2012
Hepatitis	SMAD3	P84022	0.415	0.923	0.01	2019	2019
Hepatitis	MIR215		0.559	0.654	0.01	2014	2014
Hepatitis	MIR210		0.46	0.846	0.01	2014	2014
Hepatitis	LTBP2	Q14767	0.572	0.692	0.01	2019	2019
Hepatitis	BAMBI	Q13145	0.619	0.692	0.01	2011	2011
Hepatitis	MIR106B		0.523	0.808	0.01	2017	2017
Hepatitis	PCSK9	Q8NBP7	0.482	0.808	0.01	2020	2020
Hepatitis	MIR139		0.527	0.808	0.01	2014	2014
Hepatitis	MIR143		0.462	0.885	0.01	2014	2014
Hepatitis	MIR145		0.431	0.846	0.01	2020	2020
Hepatitis	REXO1L1P	Q8IX06	0.792	0.077	0.01	1994	1994
Hepatitis	MIR196A2		0.599	0.577	0.01	2017	2017
Hepatitis	MIR200A		0.519	0.808	0.01	2018	2018
Hepatitis	MIR203A		0.471	0.808	0.01	2016	2016
Hepatitis	ARRB2	P32121	0.561	0.769	0.01	2014	2014
Hepatitis	HLA-DQA1	P01909	0.416	0.846	0.01	2008	2008
Hepatitis	APOA2	P02652	0.582	0.769	0.01	2011	2011
Hepatitis	APOB	P04114	0.453	0.808	0.01	2007	2007
Hepatitis	ERVW-1	Q9UQF0	0.505	0.692	0.01	1994	1994
Hepatitis	FFAR4	Q5NUL3	0.621	0.577	0.01	2018	2018
Hepatitis	ABCB5	Q2M3G0	0.623	0.538	0.01	2011	2011
Hepatitis	GOLGA6A	Q9NYA3	0.592	0.654	0.01	2018	2018
Hepatitis	HAS3	O00219	0.644	0.692	0.01	2013	2013
Hepatitis	HAS1	Q92839	0.67	0.577	0.01	2013	2013
Hepatitis	HABP2	Q14520	0.593	0.615	0.01	2013	2013
Hepatitis	APOC3	P02656	0.531	0.731	0.01	2017	2017
Hepatitis	TBX21	Q9UL17	0.541	0.808	0.01	2007	2007
Hepatitis	BIRC5	O15392	0.495	0.731	0.01	2011	2011
Hepatitis	UBE2K	P61086	0.631	0.654	0.01	2011	2011
Hepatitis	HLA-C	P10321	0.415	0.846	0.01	2009	2009
Hepatitis	HMGA1	P17096	0.494	0.769	0.01	2010	2010
Hepatitis	NR4A1	P22736	0.49	0.731	0.01	2017	2017
Hepatitis	HNF4A	P41235	0.461	0.808	0.01	2014	2014
Hepatitis	HNRNPA1	P09651	0.529	0.885	0.01	1997	1997
Hepatitis	HNRNPA2B1	P22626	0.566	0.808	0.01	2010	2010
Hepatitis	APBA2	Q99767	0.7	0.385	0.01	2009	2009
Hepatitis	APC	P25054	0.373	0.962	0.01	2011	2011
Hepatitis	HPGD	P15428	0.516	0.731	0.01	2017	2017
Hepatitis	AIRE	O43918	0.516	0.808	0.01	2001	2001
Hepatitis	HSF1	Q00613	0.507	0.769	0.01	2019	2019
Hepatitis	ACTBL2	Q562R1	0.539	0.769	0.01	2017	2017
Hepatitis	ICOS	Q9Y6W8	0.502	0.731	0.01	2015	2015

Hepatitis	ARSH	Q5FYA8	0.695	0.577	0.01	2018	2018
Hepatitis	GPR39	O43194	0.65	0.615	0.01	2019	2019
Hepatitis	IL4R	P24394	0.474	0.846	0.01	2018	2018
Hepatitis	HNF1A-AS1		0.621	0.538	0.01	2013	2013
Hepatitis	IL9	P15248	0.461	0.846	0.01	2018	2018
Hepatitis	IL9R	Q01113	0.659	0.654	0.01	2018	2018
Hepatitis	GNB3	P16520	0.518	0.808	0.01	2005	2005
Hepatitis	GLI2	P10070	0.446	0.846	0.01	2018	2018
Hepatitis	IL12B	P29460	0.48	0.885	0.01	2014	2014
Hepatitis	HPGDS	O60760	0.388	0.923	0.01	2010	2010
Hepatitis	GPC3	P51654	0.466	0.808	0.01	2020	2020
Hepatitis	IL17C	Q9P0M4	0.619	0.692	0.01	2017	2017
Hepatitis	STON1-GTF2A1L	Q9Y6Q2	0.682	0.385	0.01	2018	2018
Hepatitis	IL2RA	P01589	0.389	0.885	0.01	2019	2019
Hepatitis	IGF1	P05019	0.318	0.885	0.01	2018	2018
Hepatitis	GSTP1	P09211	0.383	0.923	0.01	2012	2012
Hepatitis	IGFBP7	Q16270	0.476	0.769	0.01	1996	1996
Hepatitis	IGH		0.473	0.654	0.01	2008	2008
Hepatitis	APP	P05067	0.422	0.846	0.01	2016	2016
Hepatitis	GSPT1	P15170	0.7	0.462	0.01	1987	1987
Hepatitis	GSK3B	P49841	0.43	0.846	0.01	2016	2016
Hepatitis	NR3C1	P04150	0.393	0.885	0.01	2011	2011
Hepatitis	SETD2	Q9BYW2	0.424	0.846	0.01	2017	2017
Hepatitis	GRN	P28799	0.435	0.846	0.01	2019	2019
Hepatitis	GRB2	P62993	0.546	0.769	0.01	2018	2018
Hepatitis	IL37	Q9NZH6	0.459	0.846	0.01	2017	2017
Hepatitis	ARID2	Q68CP9	0.543	0.808	0.01	2019	2019
Hepatitis	PIK3CB	P42338	0.322	0.885	0.01	2018	2018
Hepatitis	PIK3CD	O00329	0.319	0.885	0.01	2018	2018
Hepatitis	PIK3CG	P48736	0.32	0.885	0.01	2018	2018
Hepatitis	AGRP	O00253	0.488	0.769	0.01	2019	2019
Hepatitis	DPP4	P27487	0.42	0.808	0.01	2019	2019
Hepatitis	PLP2	Q04941	0.711	0.385	0.01	2008	2008
Hepatitis	PML	P29590	0.477	0.846	0.01	1998	1998
Hepatitis	DMBT1	Q9UGM3	0.533	0.654	0.01	2012	2012
Hepatitis	DLAT	P10515	0.576	0.731	0.01	1997	1997
Hepatitis	AFM	P43652	0.59	0.731	0.01	2018	2018
Hepatitis	MIR4435-2HG		0.604	0.692	0.01	2019	2019
Hepatitis	PIK3CA	P42336	0.292	0.923	0.01	2018	2018
Hepatitis	PIGR	P01833	0.615	0.808	0.01	2011	2011
Hepatitis	SIRT6	Q8N6T7	0.496	0.846	0.01	2012	2012
Hepatitis	IL23A	Q9NPF7	0.415	0.846	0.01	2017	2017
Hepatitis	TDP2	O95551	0.628	0.462	0.01	2019	2019
Hepatitis	NBAS	A2RRP1	0.566	0.692	0.01	2018	2018

Hepatitis	PGAM5	Q96HS1	0.711	0.5	0.01	2017	2017
Hepatitis	PGF	P49763	0.46	0.846	0.01	2017	2017
Hepatitis	ECHS1	P30084	0.601	0.654	0.01	2013	2013
Hepatitis	ABCB1	P08183	0.344	0.885	0.01	2012	2012
Hepatitis	ABCB4	P21439	0.529	0.615	0.01	2014	2014
Hepatitis	PHB	P35232	0.511	0.846	0.01	2020	2020
Hepatitis	ATN1	P54259	0.409	0.885	0.01	2011	2011
Hepatitis	TREM2	Q9NZC2	0.519	0.769	0.01	2017	2017
Hepatitis	TREM1	Q9NP99	0.515	0.808	0.01	2016	2016
Hepatitis	POLD1	P28340	0.507	0.769	0.01	2019	2019
Hepatitis	CYP2C9	P11712	0.506	0.885	0.01	2000	2000
Hepatitis	CYP2C19	P33261	0.467	0.846	0.01	2014	2014
Hepatitis	CYP2B6	P20813	0.428	0.846	0.01	1993	1993
Hepatitis	CYP2A6	P11509	0.556	0.731	0.01	2003	2003
Hepatitis	B2M	P61769	0.447	0.846	0.01	1993	1993
Hepatitis	CYP1B1	Q16678	0.457	0.769	0.01	2017	2017
Hepatitis	SLAMF8	Q9P0V8	0.839	0.154	0.01	2020	2020
Hepatitis	CHPT1	Q8WUD6	0.474	0.846	0.01	2016	2016
Hepatitis	CX3CR1	P49238	0.457	0.885	0.01	2019	2019
Hepatitis	PTEN	P60484	0.305	0.923	0.01	2017	2017
Hepatitis	PTGS1	P23219	0.46	0.885	0.01	2007	2007
Hepatitis	PBK	Q96KB5	0.541	0.769	0.01	2019	2019
Hepatitis	CYP3A4	P08684	0.462	0.885	0.01	2013	2013
Hepatitis	DECR1	Q16698	0.426	0.885	0.01	2019	2019
Hepatitis	KRT20	P35900	0.388	0.846	0.01	2019	2019
Hepatitis	DDX3X	O00571	0.537	0.769	0.01	2006	2006
Hepatitis	RHOF	Q9HBH0	0.638	0.5	0.01	2019	2019
Hepatitis	DDX56	Q9NY93	0.695	0.308	0.01	2006	2006
Hepatitis	CYP27A1	Q02318	0.538	0.731	0.01	2002	2002
Hepatitis	PPARG	P37231	0.358	0.885	0.01	2013	2013
Hepatitis	CYP19A1	P11511	0.41	0.885	0.01	2011	2011
Hepatitis	SARS2	Q9NP81	0.57	0.692	0.01	2008	2008
Hepatitis	RCBTB1	Q8NDN9	0.572	0.692	0.01	2018	2018
Hepatitis	MIR429		0.544	0.731	0.01	2018	2018
Hepatitis	CUX1	P39880;Q13948	0.465	0.846	0.01	2019	2019
Hepatitis	SMAD7	O15105	0.47	0.769	0.01	2019	2019
Hepatitis	MPO	P05164	0.377	0.923	0.01	2017	2017
Hepatitis	POTEKP	Q9BYX7	0.542	0.769	0.01	2017	2017
Hepatitis	MIR328		0.558	0.769	0.01	2018	2018
Hepatitis	MSD		0.674	0.654	0.01	2011	2011
Hepatitis	KDM6B	O15054	0.514	0.808	0.01	2017	2017
Hepatitis	COLGALT2	Q8IYK4	0.805	0.269	0.01	2018	2018
Hepatitis	CYTB	P00156	0.529	0.808	0.01	2018	2018
Hepatitis	MTHFR	P42898	0.337	0.885	0.01	2014	2014

Hepatitis	MTTP	P55157	0.505	0.846	0.01	2017	2017
Hepatitis	KIF1B	O60333	0.516	0.808	0.01	2012	2012
Hepatitis	MYCN	P04198	0.454	0.808	0.01	1990	1990
Hepatitis	CD200	P41217	0.529	0.769	0.01	2018	2018
Hepatitis	MMP13	P45452	0.452	0.923	0.01	2020	2020
Hepatitis	MAS1	P04201	0.533	0.769	0.01	2011	2011
Hepatitis	MBL2	P11226	0.39	0.846	0.01	2008	2008
Hepatitis	MDM2	Q00987	0.362	0.846	0.01	2009	2009
Hepatitis	FLT3LG	P49771	0.564	0.577	0.01	2017	2017
Hepatitis	MGMT	P16455	0.412	0.885	0.01	2003	2003
Hepatitis	CD99	P14209	0.491	0.731	0.01	2002	2002
Hepatitis	MIF	P14174	0.412	0.885	0.01	2006	2006
Hepatitis	CXCL9	Q07325	0.462	0.808	0.01	2015	2015
Hepatitis	MLH1	P40692	0.399	0.808	0.01	2003	2003
Hepatitis	NR3C2	P08235	0.469	0.846	0.01	1995	1995
Hepatitis	MMP9	P14780	0.305	0.923	0.01	2018	2018
Hepatitis	P2RX2	Q9UBL9	0.585	0.769	0.01	2017	2017
Hepatitis	NCAM1	P13591	0.415	0.885	0.01	2004	2004
Hepatitis	FCGR3B	O75015	0.452	0.962	0.01	2018	2018
Hepatitis	P2RY1	P47900	0.551	0.769	0.01	2017	2017
Hepatitis	P2RY2	P41231	0.543	0.808	0.01	2017	2017
Hepatitis	F9	P00740	0.465	0.885	0.01	2013	2013
Hepatitis	F2	P00734	0.415	0.885	0.01	2008	2008
Hepatitis	PC	P11498	0.515	0.808	0.01	2019	2019
Hepatitis	ESR1	P03372	0.324	0.962	0.01	2009	2009
Hepatitis	ASCC1	Q8N9N2	0.495	0.846	0.01	2018	2018
Hepatitis	NDUFA13	Q9P0J0	0.556	0.692	0.01	2017	2017
Hepatitis	CPA4	Q9UI42	0.633	0.5	0.01	2017	2017
Hepatitis	EPHA1	P21709	0.544	0.731	0.01	2011	2011
Hepatitis	MLKL	Q8NB16	0.57	0.731	0.01	2016	2016
Hepatitis	P2RX5	Q93086	0.548	0.769	0.01	2017	2017
Hepatitis	P2RX4	Q99571	0.547	0.808	0.01	2017	2017
Hepatitis	FCGR3A	P08637	0.432	0.962	0.01	2018	2018
Hepatitis	NGF	P01138	0.391	0.885	0.01	2019	2019
Hepatitis	NM		0.473	0.808	0.01	2018	2018
Hepatitis	CNOT3	O75175	0.686	0.615	0.01	2020	2020
Hepatitis	ALDH2	P05091	0.457	0.885	0.01	2019	2019
Hepatitis	OGG1	O15527	0.453	0.808	0.01	2012	2012
Hepatitis	OPRM1	P35372	0.457	0.808	0.01	2007	2007
Hepatitis	OTC	P00480	0.565	0.846	0.01	2017	2017
Hepatitis	OXA1L	Q15070	0.59	0.731	0.01	2018	2018
Hepatitis	P2RX1	P51575	0.584	0.808	0.01	2017	2017
Hepatitis	P2RX3	P56373	0.565	0.769	0.01	2017	2017
Hepatitis	PDCD1	Q15116	0.402	0.402	0.01	2011	2011
